# Genome structure and evolution of *Antirrhinum majus* L

**DOI:** 10.1038/s41477-018-0349-9

**Published:** 2019-01-28

**Authors:** Miaomiao Li, Dongfen Zhang, Qiang Gao, Yingfeng Luo, Hui Zhang, Bin Ma, Chunhai Chen, Annabel Whibley, Yu’e Zhang, Yinghao Cao, Qun Li, Han Guo, Junhui Li, Yanzhai Song, Yue Zhang, Lucy Copsey, Yan Li, Xiuxiu Li, Ming Qi, Jiawei Wang, Yan Chen, Dan Wang, Jinyang Zhao, Guocheng Liu, Bin Wu, Lili Yu, Chunyan Xu, Jiang Li, Shancen Zhao, Yijing Zhang, Songnian Hu, Chengzhi Liang, Ye Yin, Enrico Coen, Yongbiao Xue

**Affiliations:** 1 State Key Laboratory of Plant Cell and Chromosome Engineering and National Center of Plant Gene Research, Institute of Genetics and Developmental Biology, Chinese Academy of Sciences, Beijing, China; 2 University of Chinese Academy of Sciences, Beijing, China; 3 State Key Laboratory of Plant Genomics, Institute of Genetics and Developmental Biology, Chinese Academy of Sciences, Beijing, China; 4 Beijing Institute of Genomics, Chinese Academy of Sciences, Beijing, China; 5 BGI-Shenzhen, Shenzhen, China; 6 John Innes Centre, Norwich, UK; 7 National Laboratory of Plant Molecular Genetics, CAS Center for Excellence in Molecular Plant Sciences, Institute of Plant Physiology and Ecology, Shanghai Institutes for Biological Sciences, Chinese Academy of Sciences, Shanghai, China

**Keywords:** Plant sciences, Genomics

## Abstract

Snapdragon (*Antirrhinum majus* L.), a member of the Plantaginaceae family, is an important model for plant genetics and molecular studies on plant growth and development, transposon biology and self-incompatibility. Here we report a near-complete genome assembly of *A. majus* cultivar JI7 (*A. majus* cv.JI7) comprising 510 Megabases (Mb) of genomic sequence and containing 37,714 annotated protein-coding genes. Scaffolds covering 97.12% of the assembled genome were anchored on eight chromosomes. Comparative and evolutionary analyses revealed that a whole-genome duplication event occurred in the Plantaginaceae around 46–49 million years ago (Ma). We also uncovered the genetic architectures associated with complex traits such as flower asymmetry and self-incompatibility, identifying a unique duplication of TCP family genes dated to around 46–49 Ma and reconstructing a near-complete *ψS*-locus of roughly 2 Mb. The genome sequence obtained in this study not only provides a representative genome sequenced from the Plantaginaceae but also brings the popular plant model system of *Antirrhinum* into the genomic age.

## Abstract (web summary)

This study reports a near-complete genome of *Antirrhinum majus* L., provides insights into gene and genome duplication events, and reveals the genetic basis underlying complex traits of snapdragon, such as flower asymmetry and self-incompatibility.

## Main

The genus *Antirrhinum* belongs to the family Plantaginaceae and includes about 20 species with the chromosome number of 2*n* = 2*x* = 16. *Antirrhinum* originated in Europe and is mainly distributed in Europe, Asia and Africa around the Mediterranean coast. Different species in the genus *Antirrhinum* exhibit differences in flower colour, flower pattern, fragrance and flowering time; interspecific hybridization has also been described. The genus exhibits two major mechanisms that promote outcrossing: insect pollination (entomophily) and self-incompatibility^1–3^. The self-compatible *A. majus* was domesticated as a garden ornamental over 2,000 years ago^1^.

*Antirrhinum* has served as a model system for molecular and developmental genetics for the past three decades^1,4^. Several key floral genes were first identified in *Antirrhinum* including founding members of the MADS (*DEFICIENS*) and TCP (*CYCLOIDEA*) gene families, *MYB* genes controlling petal epidermal cell shape (*MIXTA*) or flower colour (*ROSEA* and *VENOSA*) and *SLF*s (*S*-*L*ocus *F*-box) controlling self-incompatibility^5–12^. Isolation and analysis of genes in *Antirrhinum* have been facilitated by the availability of endogenous active transposons^1,13^. For example, five transposable elements (*Tam1*, *Tam2*, *Tam3*, *Tam4* and *Tam11*) ^14–18^ have contributed to the identification of floral homeotic genes. However, so far these studies have been carried out without the benefit of a genome sequence to provide an overall evolutionary and architectural context for these genes, transposons and traits.

Here we report a near-complete genome assembly of *A. majus*. We annotated 37,714 protein-coding genes on the basis of expression and homology evidence. The assembly was generated by combining whole-genome shotgun (WGS) sequencing of short reads on the Illumina platform and single-molecule real time (SMRT) long reads on the Pacific Biosciences (PacBio) platform. Most of the assembled sequences were anchored onto chromosomes to form eight pseudomolecules using a genetic map. Comparative analysis based on this sequence reveals that the Plantaginaceae and Solanaceae diverged from their most recent ancestor about 62 Ma, and that a whole-genome duplication (WGD) event occurred around 46–49 Ma. We found that the WGD contributed to the evolution of the TCP gene family related to flower asymmetry in *A. majus*. We also analysed the near-complete genomic structure of the pseudo (*ψ*) *S*-locus of *A. majus* of roughly 2 Mb, which contained 102 genes including 37 SLF genes. The genome sequence provided here will accelerate genomic and evolutionary studies in this model species.

## Results

### Genome sequencing, assembly and annotation of *A. majus*

**Fig. 1:**
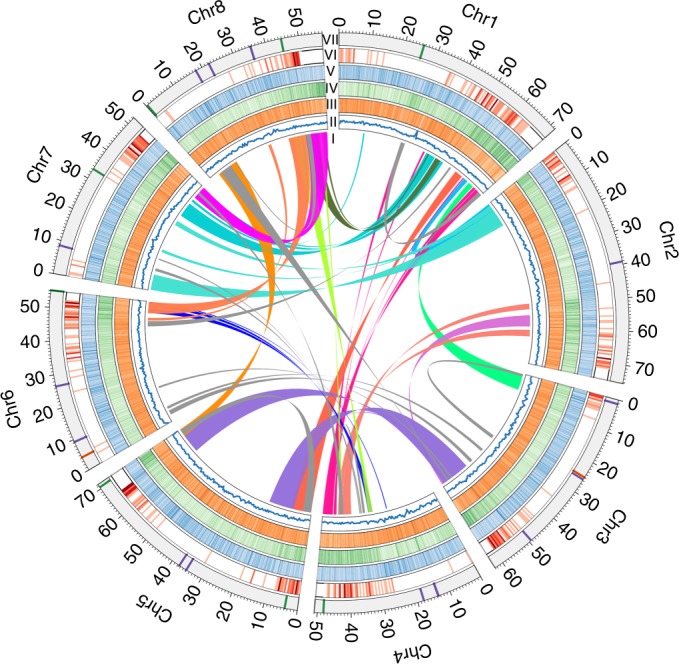
An overview of the genomic features of *A. majus* JI7. Roman numerals refer to: I, duplications of genomic paralogous sequences; II, guanine-cytosine (GC) content; III, simple sequence repeats; IV, gene density; V, retroelement density; VI, recombination rate (deep colour shows high recombination rates) and VII, eight *Antirrhinum* chromosomes with physical distances including low copy number repetitive elements: telomere repeat *TTTAGGG* (green), 5S recombinant DNA (orange) and pericentromeric repeats *CentA1* and *CentA2* (purple). A ruler with marks every 1 Mb is drawn on each chromosome.

We sequenced a highly inbred *Antirrhinum* line (*A. majus* cv. JI7) using a combination of Illumina short-read and PacBio long-read sequencing technologies. The genome size was estimated from *k*-mer distributions to be about 520 Mb. We obtained 90.85 gigabases (Gb) high-quality Illumina paired-end reads, equivalent to 174-fold sequence coverage of the genome. We used CANU^19^ to correct and assemble 25.89 Gb PacBio reads into contigs and SSPACE^20^ for scaffolding with Mate-paired short reads. The assembled genome size was 510 Mb with contig and scaffold N50 (the size above which 50% of the total length of the sequence assembly can be found) sizes of 0.73 and 2.6 Mb, respectively (Fig. 1, Table 1, Supplementary Fig. 1 and Supplementary Table 1–4). The mapping ratio of ×42.22 Illumina data was 99.55% (Supplementary Table 5) and the coverage of contigs using PacBio data was 99.97%. The heterozygosity of the assembled genome was estimated to be 51 single nucleotide polymorphs (SNPs) per 1 Mb (0.0051%) by using Illumina sequencing data.

**Table 1:** Statistics for the *Antirrhinum* genome and gene annotation

Estimate of genome size	520 Mb
GC content	35.50%
N50 length (contig)	0.73 Mb
Longest contig	3.74 Mb
Total size of assembled contigs	510.20 Mb
N50 length (scaffold)	2.62 Mb
Longest scaffold	9.90 Mb
Total size of assembled scaffolds	511.70 Mb
Number of genes	37,714
Average gene length	3,166 bp
Gene density	73.95 Mb^−1^
Transcripts number	52,780
Average coding sequence length	1,036 bp
Average protein length	344 amino acids
Average exon length	245 bp
Average intron length	314 bp
Tandem repeat	13.03 Mb

To anchor the *A. majus* genome sequence to chromosomes, we created linkage maps by re-sequencing 48 recombinant inbred lines (RILs) derived from *A. majus* crossed to the self-incompatible species *A. charidemi*. We identified a total of 4,523,444 homozygous SNPs between the parents on 1,386 contigs and obtained 4,198,995 SNPs on 1,381 contigs for linkage map construction. After validations by known genetic markers^21,22^, 496.9 Mb (97.12%) of the assembled scaffold sequences were anchored onto eight linkage groups to form pseudomolecules. The pseudomolecules ranged in size from 50.9 to 75.4 Mb. The average recombination rate was 1.798 centimorgans per Mb (Supplementary Table 6 and Supplementary Data Set 1). The relationship between genetic and physical distances revealed significantly lower recombination rates at the centromere regions of all chromosomes and the extended pericentromeric regions of chromosomes 4, 6, 7 and 8. The linkage groups were linked to the physical chromosomes through fluorescence in situ hybridization (FISH) (Supplementary Fig. 2–4 and Supplementary Table 6).

To evaluate the assembled genome quality, we aligned 25,651 expressed sequence tags (ESTs) of *Antirrhinum* downloaded from National Center for Biotechnology Information (NCBI) nucleotide database to the assembled genome, and found that 96.59% of the ESTs could be mapped. Alignments between the assembled genome and three sequenced Bacterial Artificial Chromosomes (BACs) indicated an average nucleotide accuracy of 99.65% in the assembly. BUSCO^23^ analysis showed 93.88% complete genes at the genome mode and 93.40% at the protein mode, which suggested that the quality of the assembled *Antirrhinum* genome sequence was comparable to that of other published plant genomes (*Petunia* and *Arabidopsis*) (Supplementary Fig. 5–7 and Supplementary Data Set 2). Taken together, these results suggested that the *A. majus* genome assembly was both highly accurate and near completion.

We predicted a total of 37,714 protein-coding genes with an average transcript length of 3,166 base pairs (bp) by using a combination of ab initio and evidence-based methods^24^. We used *Antirrhinum* EST sequences and RNA-seq data from six major tissues: leaf, root, stem, stamen, pistil and pollen (Supplementary Data Set 3) to confirm the expression of the genes. Approximately 89% of the genes were functionally annotated. The average gene density in *Antirrhinum* was one gene per 15.5 kilobase (kb), which is about three times lower than *Arabidopsis* (one gene per 4.5 kb) and slightly higher than tomato (one gene per 25.7 kb). Genes were distributed unevenly, being more abundant towards the ends of the chromosomal arms (Fig. 1). We identified genes encoding 981 transfer RNAs, 800 microRNAs, 10 ribosomal RNAs (18S, 28S, 5.8S and 5S) and 622 small nuclear RNAs. A total of 268.3 Mb (52.6%) of sequences was annotated as repeats, including a wealth of class I (retrotransposon: 182.8 Mb) and class II (DNA transposon: 41.1 Mb) elements (Supplementary Tables  7–10).

We found 95 transposable elements belonging to the *En*/*Spm*/*CACTA* family. Three subfamilies (*Tam2*, *Tam4* and *Tam11*) had copies with 100% identity, suggesting recent duplication/transposition events. We also identified 166.21 Mb comprising long-terminal repeat (LTR) retrotransposons, with sequence similarity between copies indicating a mean divergence time of ~0.86 Ma. Bursts of *Gypsy* and *Copia* retrotransposon insertions were detected at 0.1–0.2 Ma and 120–130 Ma, respectively. These results suggest that the *Antirrhinum* genome has a long history of active transposition (Supplementary Table 11, Supplementary Fig. 8 and Supplementary Data Set 4).

### Comparative genomic analysis of *A. majus*

**Fig. 2:**
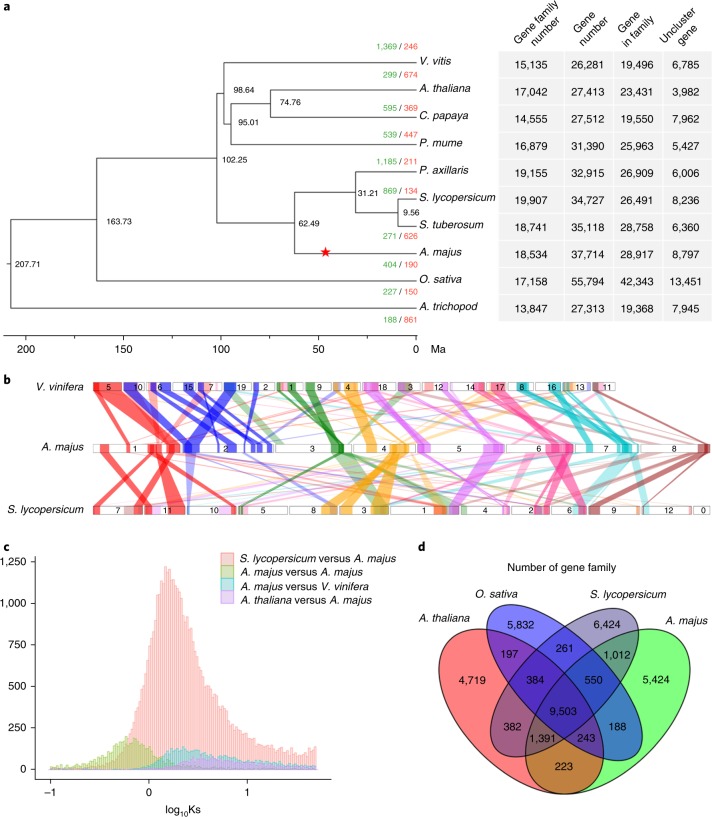
Genome evolution of *A. majus*. **a**, Phylogenetic tree of angiosperm species including their divergence time on the basis of orthologues of single-gene families. The red star highlights the genome duplication in the *A. majus* lineage. The number in each node indicates Ma between two divergent branches. Green/red numbers indicate expansion and contraction gene families. *A. trichopoda* was used as an outgroup. Bootstrap values for each node are above 100%. **b**, Synteny blocks among chromosomes of *A. majus*, *V. vinifera* and *S. lycopersicum*. The numbers represent individual chromosomes. The selected syntenic gene numbers are more than 50 in each block. **c**, Density distributions of Ks for paralogous genes among *A. majus*, *V. vinifera* and *S. lycopersicum*. **d**, A Venn diagram of shared orthologues among four species of *A. majus*, *S. lycopersicum*, *A. thaliana* and *O. sativa*. Each number represents a gene family number shared among the genomes.

Self-alignment analysis revealed duplicated and triplicated regions between and within chromosomes. Paralogous relationships among the eight *Antirrhinum* chromosomes revealed 45 major duplications and two triplications, collectively containing 1,841 pairs of paralogous genes (Fig. 1 and Supplementary Data Set 5). We performed all-against-all comparisons to identify 2,115 single-copy genes of *Antirrhinum* with orthologues in nine angiosperm species (*A. majus*, *Arabidopsis thaliana*, *Amborella trichopoda*, *Carica papaya*, *Oryza sativa*, *Petunia hybrida*, *Prunus mume*, *Solanium lycopersicum*, *Symphytum tuberosum* and *Vitis vinifera*). The resulting phylogenetic tree (Fig. 2a) show that the *Antirrhinum* lineage split from potato and tomato lineages around 62 Ma, consistent with the results of Bell et al.^25^.

For inter-species comparative genomic studies, we examined the synteny of *Antirrhinum* chromosomes and those of *V. vinifera* and *Solanum lycopersicum*. We found only small syntenic blocks between the *A. majus* and the *V. vinifera* or *S. lycopersicum* chromosomes (Fig. 2b). We also compared *A. majus* with *Sesamum indicum*, *Olea europea*, *Helianthus annuus* and *Coffea arabica*, which all belong to Lamiales. Large syntenic blocks were found between the *Antirrhinum* genome and these species, especially between *Antirrhinum* chromosomes 1, 2, 6 and 8 and *C. arabica* chromosomes 3, 1/1, 4/7, and 6, respectively (Supplementary Figs. 9 and 10).

We identified the syntenic blocks within the *A. majus* genome through intragenome comparisons. We calculated the density distribution of synonymous substitution rate per gene (Ks) between the collinear paralogous genes and inferred paleotetraploidy event in *Antirrhinum*. A peak at around 0.57–0.60 indicated that a WGD, which was Plantaginaceae-specific, occurred around 46–49 Ma (Fig. 2c), clearly after the divergence of *Antirrhinum* lineage from the lineage of potato and tomato.

We then compared the complexity of gene families between *Antirrhinum* and other species: 9,503 gene families were shared by *Antirrhinum*, *Arabidopsis*, rice and tomato; 6,677 gene families were possibly contracted in *Antirrhinum*, while the other 3,778 gene families were expanded (Fig. 2d). Gene-set enrichment analysis (GSEA) analysis results showed that gene families encoding protein kinase activity, catalytic activity, transporter activity and ATP-binding activity were most obviously expanded (Supplementary Table 12). Transcription factor gene families, such as AP2, C2H2, GRAS, TCP and Trihelix, were expanded in species belonging to the order Lamiales (*A. majus*, *S. indicum* and *O. europea*) compared with *A. thaliana*, *S. lycopersicum* and *V. vitis* (Supplementary Table 13 and Supplementary Data Set 6).

We analysed the contributions of tandem duplication and WGD to expansions/contractions. We found most F-box domain (PF00646), cytochrome P450 (PF00067) and NB-ARC domain (PF00931) gene families were derived from tandem duplication events (percentage of tandem duplication genes >40%, compared with the genome average 12.1%). The percentage of expanded transcription factor genes caused by WGD (27.5%) was larger than the genome average (16.4%), especially for WRKY (49.2%), MADS-MIKC (43.3%), bZIP (39.1%), TCP (37.5%) and G2-like GARP (36.8%) (Supplementary Data Sets 6 and 7). Thus, both WGD and tandem duplication have played an important role in the expansion of gene families.

### Evolution of floral asymmetry and TCP family

**Fig. 3:**
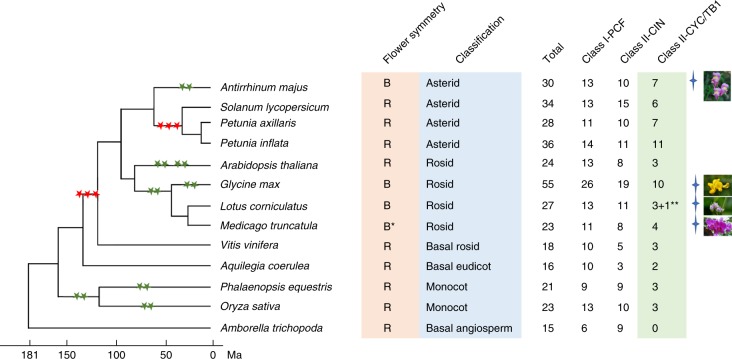
Evolution of flower symmetry and TCP gene family. Left, a phylogenetic tree of the flowering plants derived from their divergence time based on orthologues of single-gene families. Three red stars show the whole-genome triplication and two green stars the duplication events (http://chibba.agtec.uga.edu/duplication/). Right, B represents bilateral flower symmetry and R radial flower symmetry; Asterid, Rosid, Basal rosid, Basal eudicot, Monocot and Basal angiosperm represent the clade names, respectively. Total numbers of TCP family genes, Class I PCF, Class II CIN and Class II CYC/TB1 are shown from left to right. The asterisk indicates the sequenced genome of species of *Medicago truncatula* with flower radial symmetry, but flowers of most *Medicago* species have bilateral symmetry. The double asterisk indicates *Lotus corniculatus* in which three TCP genes were identified but a functional TCP gene was not detected in its genome. Four-pointed stars denote flowers with bilateral symmetry with their photos from PPBC (http://www.plantphoto.cn).

*A. majus* has served as the genetic model of floral symmetry. Previous studies have revealed that floral asymmetry in *A. majus* is largely controlled by two transcripion factors (TFs) (*CYC* and *DICH*) that belong to the TCP gene family^7,8,26^. To explore their evolution, we analysed the composition of the TCP families in *A. majus* and several sequenced angiosperms with floral symmetry. The TCP family is divided into two classes, class I (PCF) and II, and class II is further divided into two clades, CIN and CYC/TB1. Both eudicot and monocot share a subfamily containing *CYC* and *DICH* genes that belong to the CYC/TB1 clade. However, the basal angiosperm *A. trichopoda*, which has radially symmetrical flowers, lacks any members of the class II CYC/TB1 clade. Two monocots and several eudicots also have radially symmetrical flowers (Fig. 3). These findings suggest that the TCP class II CYC/TB1 clade^26,27^ appeared after the emergence of radially symmetrical flowers, and the initial role of CYC/TB1 clade was thus not likely to be involved in the control of floral symmetry (Fig. 3 and Supplementary Data Set 8).

We identified a total of 30 putative functional TCP family genes in *Antirrhinum*: 13 class I genes and 17 class II genes (10 in the CIN clade and 7 in the CYC/TB1 clade) (Supplementary Data Set 8). Syntenic block and Ks analyses of the orthologous gene pairs revealed that both WGD and tandem duplication contributed to the expansion of TCP family members. A previous study showed that *CYC* and *DICH* have partial redundancy in the control of flower asymmetry and exhibit only partially similar expression patterns in floral meristems in *A. majus*, and the two genes act together to establish the flower asymmetry in *A. majus*^8^. We found that the *CYC* and *DICH* genes reside on a pair of syntenic regions including 79 homologous gene pairs (Supplementary Table 14). The Ks analysis results show that this syntenic block was retained from the Plantaginaceae-specific WGD event. Previous phylogenetic analysis suggested that zygomorphic flowers independently evolved from actinomorphic ancestors more than 25 times^28^. On the basis of fossil records, it was proposed that clearly zygomorphic flowers emerged in various lineages roughly 50 Ma^29^, concurrent with the occurring time of the WGD event. These results suggest that the WGD to generate both *CYC* and *DICH* genes played a critical role in the evolution of zygomorphic flowers in the *Antirrhinum* lineage.

Furthermore, two MYB-class genes *RAD* and *DIV*, acting downstream of *CYC/DICH* in the control of floral symmetry, interact with the *DRIF* gene. The *DRIF* has homologous copies with similar Ks to *CYC*/*DICH*, and they are also located at a WGD-derived syntenic block ^30-32^. These results further support the idea that the key regulators of floral asymmetry were retained from the genes generated by the WGD in *Antirrhinum*.

### Structure of the *ψS*-locus in *A. majus* and its gene collinearity in self-incompatible species

**Fig. 4:**
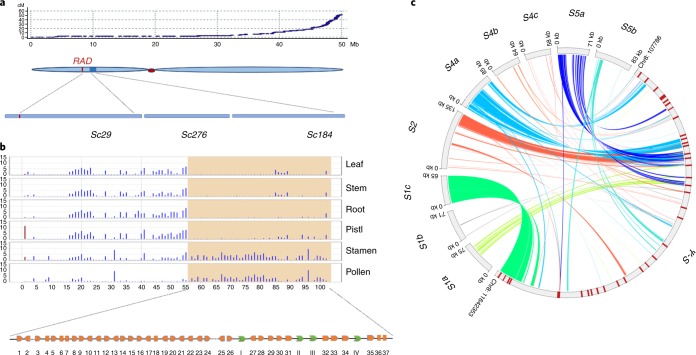
Genomic features of the *ψS*-locus of *A. majus* and its synteny with the *S*-locus regions of *A. hispanicum*. **a**, Chromosomal locations of three scaffolds covering the *ψS*-locus region of *A. majus*. A genetic recombination map of chromosome 8 is shown on the top panel. The *x* axis shows its physical distance (Mb) and the *y* axis its genetic distance (cM). A schematic representation of chromosome 8 is shown in the middle panel with a red dot indicating its centromere. The *ψS*-locus is depicted as a blue box on its short arm. A vertical red line in the chromosome indicates the *RAD* gene. The lower panel shows three scaffolds of *Sc29*, *Sc276* and *Sc184* covering the *ψS*-locus region. **b**, Transcriptional profiles of the *ψS*-locus and its flanking regions of *A. majus*. The light orange shadow denotes the predicted *ψS*-locus region (*SLF1*–*SLF37*). This region between *RAD* and *SLF37* contains a total number of 102 annotated genes. The bottom panel is a schematic representation of the *SLF* genes. Orange squares indicate the *ψSLF* genes and green arrows the other annotated genes (I: a putative MYB family transcription factor; II and III, putative RNA-binding proteins and IV, a putative phosphate-dependent transferase). **c**, The synteny of the *S*-locus regions between *A. majus* and *S*_*1*_, *S*_*2*_, *S*_*4*_ and *S*_*5*_ haplotypes of *A. hispanicum*. Different colours indicate syntenic and inversion regions between the *ψS*-locus and *S*_*1*_ (*S*_*1a*_, *S*_*1b*_ and *S*_*1c*_), *S*_*2*_, *S*_*4*_ (*S*_*4a*_, *S*_*4b*_ and *S*_*4c*_) or *S*_*5*_ (*S*_*5a*_ and *S*_*5b*_) haplotypes of *A. hispanicum*.

In previous cytological investigations, we found that the *Antirrhinum S*-locus is located in a heterochromatin region on the short arm of chromosome 8 (ref. ^33^). The cultivated species *A. majus* is self-compatible, carrying a pseudo (*ψ*)*S*-locus. Scanning the *A. majus* genome for conserved (FBA/FBK domain) of the *SLF* gene family revealed the presence of 37 *SLF* genes (*SLF1*–*SLF37*) located in the short arm of chromosome 8, probably corresponding to the *ψS-*locus. The locus covered 874 kb across three scaffolds *Sc29*, *Sc276* and *Sc184*, possessing a considerably higher number of *SLF* genes than *S. lycopersicum*^34,35^ (Fig. 4a). Six pseudogenes with FBA domains were inferred to be loss-of-function. No S-RNase was found in or near the locus, suggesting it might have been lost during horticultural selection for self-compatible *A. majus*. The *RAD* gene was located about 1 Mb upstream of *SLF1*, consistent with previous studies showing its linkage with the *S*-locus^36^. Expression analysis showed that 30 *SLF* genes were expressed in either pollen or anthers indicating they could play a role in pollen function. The number of *SLF* genes in the *A. majus ψS-*locus is more than twice that found in *S. lycopersicum* (15 *SLF* genes including 11 pseudogenes)^33,35^ and that the *ψS*-locus of *A. majus* contains the largest number of active *SLF* genes annotated so far in a plant genome (Fig. 4b and Supplementary Data Sets 9–11).

We compared the *ψS*-locus sequence with nine assembled TAC (transformation-competent artificial chromosome) sequences from four *S* haplotypes of self-incompatible *A. hispanicum*. Gene collinearity between *S*-alleles was revealed in the genomic region extending from *AhSLF12* to *AhSLF13* (Fig. 4c). An intrachromosome inversion around the *S*-locus was found to occur in the *S*_*2*_ haplotype of *A. hispanicum* as described previously^33^. In contrast to the *ψS*-locus of *A.majus*, an *S-RNase* gene was found in every sequenced *A. hispanicum S* allele, suggesting that the *S-RNase* had been deleted in the *ψS*-locus. Notably, a pseudo-gene *AmSLF18* in the *A. majus ψS*-locus had an orthologue in the *S*_*4*_ haplotype in *A. hispanicum*, which had a complete coding sequence and was expressed, suggesting the latter is an active gene in the *S*-locus and the former lost function, possibly following the loss of *S*-RNase (Fig. 4c and Supplementary Data Set 11). The orthologous *SLFs* among different haplotypes show a low allelic diversity, consistent with our previous finding^34^.

The nonsynonymous (Ka) and synonymous (Ks) substitution rates of the 12 collinear *SLF* gene pairs showed that the values of *SLFs* are lower than that of *S-RNase* in *Antirrhinum*, and the allelic *SLF* genes showed a ratio of Ka/Ks = 0.41, consistent with a negative frequency-dependent selection detected previously^35,37^. Only *SLF14* appears to be a positively selected gene (Ka/Ks > 1) (Supplementary Data Set 12). The average divergence time of these orthologous *SLF* genes was estimated to be 4 Ma, similar to an estimated early *Antirrhinum* species divergence time of less than 5.3 Ma^38^. However, the average divergence time of the *S-RNases* of *A. hispanicum* is estimated to be around 62–120 Ma, similar to the species divergence between *Antirrhinum* and Solanaceae species estimated in our study (Supplementary Data Set 13). These results suggest that the divergence of S-RNase occurred before the WGD and they were well maintained in the lineage of *Antirrhinum*. Our results showed that a near-complete *ψS*-locus *A. majus* was identified.

## Discussion

The genome sequence of *A. majus* reported here represents a sequenced genome of a species belonging to the family Plantaginaceae, and reveals a WGD specific to this family. Zygomorphy in the *Antirrhinum* lineage is thought to have arisen in the late Cretaceous period in the fossil record^38^, as a mechanism facilitating insect-mediated pollination. We show that two key TCP genes controlling zygomorphy are collinear on a syntenic block generated by the WGD of the Plantaginaceae, suggesting that the duplication may have provided a genetic basis for the evolution of this trait. An independent WGD may have been involved in the evolution of zygomorphy in *Glycine*^39^, and the missing type I Mβ MADS-box genes family resulted in bilaterally symmetrical flowers in the Orchidaceae^40^. The timing of the WGD event in the Plantaginaceae corresponds to the age of this family on the basis of the molecular dating approaches^25^. WGD events have been considered to be catalysts for species diversification and evolutionary novelty in plants^41–43^. The availability of more species with completely sequenced genomes in the Plantaginaceae and their relatives may help clarify the role of the WGD in the expansion and evolution of the family.

The *Antirrhinum* genome also sheds light on the evolution of self-incompatiblity. The fine genomic structure of the *ψS*-locus from *A. majus* reveals a large number of pollen *SLFs*, probably due to gene duplication, recombination suppression, purifying selection and frequency-dependent selection associated with the *S*-locus^35,37^. Relatively low allelic diversity was observed between orthologous *SLFs* among different haplotypes^34^, compared with the paralogues within a haplotype, perhaps because extensive divergence would lead to recognition and self-inactivation of S-RNase resulting in loss of self-incompatibility. The deletion of S-RNase in cultivated *A. majus* could be responsible for the loss of self-incompatibility, giving an essentially irreversible transition. Such deletions may account for why self-compatible species are difficult or almost impossible to revert back to self-incompatible species (Doll’s Law)^44^. The high microcolinearity of the *S*-locus between self-incompatible and self-compatible *Antirrhinum* indicates that the deletion of *S-RNase* in self-compatible species was a recent event. In fact, some mutated *SLF* genes in different haplotypes also arose recently^34^.

The physical size of the *S*-locus in *S. lycopersicum* is much larger than that in *A. majus* (17 Mb compared to 2 Mb)^36,45^, yet it contains fewer *SLF* genes (17 compared to 37). Less repetitive sequences are found in the *ψS*-locus and *S* loci of *Antirrhinum* compared with that of *Solanum*, suggesting that an increase of the gene numbers through unequal crossovers possibly results in the *Antirrhinum S*-locus, and that repetitive element enrichments could underlie the large physical size and low gene density of the *S*-locus of *Solanum*, enhanced perhaps by its centromeric location.

In conclusion, the assembled *A. majus* sequence provides a reference genome for the Plantaginaceae and will be helpful for genetic, genomic and evolutionary studies in both *Antirrhinum* and other flowering plants. For example, studies on a natural hybrid zone between *Antirrhinum* species using this genome sequence as a reference have revealed patterns of selection and gene flow underlying the evolution of flower colour pattern^46^. We hope the resource will be a useful stimulus to further studies.

## Methods

### Plant materials

The seeds of cultivated *Antirrhinum* (*A. majus* JI7) were surface-sterilized and plated on Murashige–Skoog (1/2 MS) plates (×1/2 MS salts, 0.23% phytagel and ×1 Gamborg’s B5 vitamin mixture, all from Sigma) and grown in growth chambers (160 h/8 h, light/dark) with white fluorescent light (100 μmol m^−2^ s^-1^) at 22 °C. After avoiding light for 72 h, we harvested leaf tissues and extracted DNA using the cetyltrimethylammonium bromide (CTAB) method^47^.

To generate the RILs, *A. majus* JI7 stock (TA7–7) was crossed to *A. charidemi* (TA1282). The *A. charidemi* individual was derived from accession Ac1024 -Y-TES -1, with seed collected from Cabo de Gata Spain. A single F_1_ hybrid (P107-2) was self-pollinated to produce a total of 195 F_2_ plants. Each plant was self-pollinated to produce a unique RIL. A total of 48 RILs were eventually developed from single F_2_ individuals taken through additional rounds of self-pollination through to the F_7_ to F_9_ generation. *A. hispanicum* lines (*AhS*_*2*_*S*_*4*_ and *AhS*_*1*_*S*_*5*_) were maintained by vegetative cuttings as described by Xue et al.^48^. and were originally sourced from the Gatersleben collection (http://www.ipk-gatersleben.de/en/gbisipk-gaterslebendegbis-i/).

### WGS

High-quality genomic DNA was extracted from young leaves of cultivated *A. majus* JI7 using the CTAB method. According to the manufacturer’s instructions (Illumina HiSeq 2000), we constructed a total of 2 × 100 paired-end sequencing libraries with insert sizes from 170 bp to 20 kb for standard WGS sequencing. For small-insert (<2 kb) libraries, DNA was fragmented, end repaired, ligated to Illumina paired-end adaptors, size selected and purified by PCR amplification. For large-insert (≥2 kb) mate-paired libraries, about 20–50 μg genomic DNA was fragmented, and biotin-labelled adaptors were annealed to the fragment ends before self-ligation to form circularized DNA. This library was re-fragmented and target sequences (that is, the long molecule ends) were enriched using biotin/streptavidin, and then prepared for sequencing. All of the above libraries were sequenced on Illumina Genome Analyzer sequencing platforms. In total, we generated about 90.85 Gb (roughly ×144.24) reads. Using the Pacific Biosciences (PacBio) platform for single-molecule, real-time (SMRT) sequencing we generated a total of 25.89 Gb from 30 SMRT cells, with an average subread length of 5.2 kb and a N50 size of 13.4 kb. The 48 individual RILs were genotyped using the WGS sequencing. We obtained a total of 201.49 Gb sequencing data and the average sequencing depth of each sample was 4.5 Gb (×8.2). 92.40% of the reads could be mapped into the genome.

The genome size was evaluated using the total length of sequence reads divided by sequencing depth as described^49^. To estimate the sequencing depth, we counted the frequency of each 17-mer from the Illumina WGS sequencing reads and plotted the distribution of copy numbers. The peak value of the frequency curve represents the overall sequencing depth. We used the algorithm (*N* × (*L* − *K* + 1) − *B*)*/D* = *G*, where *N* is the total sequence read number, *L* the average length of sequence reads and *K* the length defined as 17–31 bp here. To minimize the influence of sequencing error, *K*-mers with low frequency (<4) are discarded. *B* is the total number of low-frequency *K*-mers. *G* denotes the genome size and *D* is the overall depth estimated from *K*-mer distribution.

### Genome assembly

The assembly was performed on HPC (High Performance Computing) system with 40 nodes, each one having 16 CPU cores and 128 GB of RAM. The operating system was Centos 6.3 64-bit (Linux). We corrected the PacBio long reads using the Canu pipeline^19^, and then assembled them into contigs (N50 = 733 kb; total length = 510 Mb). The Canu pipeline parameters were: genome size = 600 Mb, error rate = 0.013. We then further polished the PacBio assembled contigs using Quiver^49^. We used the mate-pair sequences to connect the contig sequences with SSPACE^20^. Initially, we required 30 connections to support connection of contig sequences into a scaffold. We then repeated this process iteratively using the result of the scaffolding as input but reducing the connection support by five. The final assembly spans were produced with the connection support parameter set to 10.

To construct the linkage map and organize scaffolds into pseudochromosomes, we resequenced individual RILs and their parents. The raw reads generated from the Illumina-Pipeline included low-quality, adaptor contaminated and duplicated reads. Reads were filtered using Trimmomatic^50^ with default parameters, retaining only reads longer than 50 bp after quality trimming. We used BWA-mem^51^ (http://bio-bwa.sourceforge.net/) with default settings to align filtered reads to the assembled genome. After alignment, we used SAMtools^52^ to filter out low-quality (mapping quality <30) alignments and the Genome Analysis Toolkit (GATK)^53^ (http://www.broadinstitue.org/gatk/) UnifiedGenotypers to call SNPs. The SNPs were filtered using the GATK VariantFiltration program with the following criteria: clusterWindowSize:10, MQ0>=4& ((MQ0/ (1.0* DP)) >0.1), QUAL<50.0, DP< 5. A total of 4,523,444 homozygous SNPs were identified between the parents on 1,386 contigs and used to filter out unmatched SNPs or extremely unevenly distributed SNPs in the RIL population. A total of 4,198,995 filtered SNPs and 2,300 bin markers were obtained on 1,381 contigs for the linkage map construction. Published markers^19,20^ were used to validate the linkage map (Supplementary Table 5 and Supplementary Data Set 1). Fifty SNP sliding windows were used to create SNP bins to find recombination sites. JoinMap4.1 (https://www.kyazma.nl/index.php/JoinMap/) ML methods were used to cluster the bins into LGs, and then the MstMap^54^ (http://www.mstmap.org/download.html) Kosambi model was used to compute the order of the bins. The final map anchored 1,280 contigs to eight linkage groups.

To evaluate the assembled genome quality, first we mapped the illumina NGS data to the genome using BWA-mem^51^ (http://bio-bwa.sourceforge.net/). Then we aligned the EST sequence download from NCBI (http://www.ncbi.nlm.nih.gov/nucest/?term=EST%20Antirrhinum) using BLAT^55^. Finally, we used BUSCO^23^ (http://busco.ezlab.org, v3) to examine the gene content with Embryophyta odb9 database and parameters. We also used BWA-mem^51^ with default settings to align three BAC sequences to the assembled genome. GenBank numbers of the three BACs are AY935269.1, FJ404769.1 and FJ404770.1 with lengths of 85, 51 and 111.3 kb, separately.

### Gene structure annotation and functional annotation

The gene annotation in the *A. majus* genome was performed by a combination of ab initio and evidence-based methods^24^. The protein sequences from three sequenced plants, namely, *A. thaliana*, *C. papaya* and *S. tuberosum*, were aligned to the genome using TBLASTN^55^ with an *E* value cut-off of 1 × 10^–5^. The homologous genomic sequences were aligned against the matching proteins using GeneWise (https://www.ebi.ac.uk/Tools/psa/genewise/)^56^ for accurate spliced alignments. For ab initio prediction, Augustus (http://augustus.gobics.de)^24^ and GlimmerHMM^57^ were run on the repeat masked genome with parameters trained from the closely related species and partial or small genes that had less than 150 bp coding length were discarded. EST was aligned to the genome using BLAT^58^ to generate spliced alignments, which were linked according to the overlap using PASA^59^. Finally, we aligned all the RNA reads to the reference genome by TopHat^60^ (https://ccb.jhu.edu/software/tophat/index.shtml), assembled the transcripts using Cufflinks^61^ under default parameters and predicted the open reading frames to get reliable transcripts with HMM-based training parameters. To finalize the gene set, all the predictions were combined using GLEAN^62^ to produce the consensus gene sets. On the other hand, another gene annotation in the snapdragon genome was performed using the Gramene pipeline^63^. The evidence included 167 messenger RNAs and 25,310 ESTs of *Antirrhinum* from the NCBI nucleotide database (https://www.ncbi.nlm.nih.gov), and SwissProt proteins for plants, which were cleaned up by removing redundant sequences with a minimum threshold of 80% for both identity and coverage, which left us with 340,312 sequences. Meanwhile, the mRNAs and ESTs of eudicot species were downloaded from NCBI and filtered to remove redundant sequences with a cut-off of 90% for both identity and coverage, resulting in 2,332,979 complementary DNAs and 152,396 ESTs, and RNA-seq data from six samples of *A. majus* were downloaded in this study and assembled into contigs using SOAPdenovo-trans v.1.03 (http://soap.genomics.org.cn/SOAPdenovo-Trans.html). The assembled contigs were used as same-species EST evidence. The genes with protein length <100 amino acids and expression level in RNA-seq data <1 RKPM were discarded. In the end, the predicted genes were obtained after selecting the longer ones between overlapping genes.

Gene functions were assigned according to the best match derived from the alignments to the integral database consisting of annotated proteins in *Arabidopsis* (https://www.*arabidopsis*.org/download_files/Proteins/TAIR10_protein_lists/TAIR10_pep_20101214) databases and SwissProt proteins using BLASTP^55^, with 30% minimum identity and coverage as threshold. We annotated motifs and domains using InterProScan^64^ by searching against publicly available databases, including ProDom^65^ (http://prodom.prabi.fr/), PRINTS^66^ (www.bioinf.manchester.ac.uk/dbbrowser/PRINTS/), Pfam^67^ (http://pfam.xfam.org/), SMART^68^ (http://smart.embl-heidelberg.de/), PANTHER^69^ (http://www.pantherdb.org/), SUPERFAMILY^70^ (http://supfam.org/SUPERFAMILY/), PIR (http://pir.georgetown.edu/) and PROSITE (http://prosite.expasy.org/). Both CPC program^71^ and gene prediction evidence such as poor coding ability and protein length were used to filter the non-coding genes. All data for the evidence-based prediction were downloaded from corresponding databases on 5 January 2017, with the minimal length of 150 bp per 50 amino acids.

The *tRNA* genes were identified by tRNA scan-SE^72^ (https://wiki.gacrc.uga.edu/wiki/TRNAscan-SE) with eukaryote parameters. For rRNA identification, we aligned the *A. thaliana* rRNA sequences against the *A. majus* genome by using BLASTN^55^ with an *E* value of 1 × 10^-5^. The snRNA and miRNA predictions were made using INFERNAL^73^ software (http://eddylab.org/infernal/) and by searching against the Rfam^74^ database (http://rfam.xfam.org/).

The classification of genes into families was carried out by BLASTP^55^ all-against-all comparisons of predicted proteins using the duplicate_gene_classifier module integrated within MCScan^75^ (http://chibba.pgml.uga.edu/mcscan2/) with default parameters. The MCScan software classified the duplicate genes of one genome into whole genome /segmental (≥5 homologous gene pairs in collinear blocks), tandem (consecutive repeat), proximal (in nearby chromosomal region but adjacent within 10 genes) or dispersed (modes other than segmental, tandem and proximal) duplications. Remaining genes were defined as singletons. The iTAK^76^ database (http://itak.feilab.net/cgi-bin/itak/index.cgi) was used to analyse transcription factor family expansion and to assign genes to specific families.

### Genome repeat element identification

Repetitive sequences and transposable elements in the genome were identified using a combination of de novo and homology-based approaches at both the DNA and protein levels. Briefly, we first constructed a de novo repeat library for snapdragon by using LTR_FINDER^77^ (http://tlife.fudan.edu.cn/ltr_finder/) and passed this to Repeat Modeler^78^ v.1.08 with default parameters. This library was aligned to the PGSB Repeat Element Database (http://pgsb.helmholtz-muenchen.de/plant/recat/) to generate the classification information for each repeat family. For identification of transposable elements at the DNA level, RepeatMasker was applied using both the repeat database we had built and Repbase^79^ (http://www.girinst.org/repbase). Next, we executed RepeatProteinMask^78^ (http://www.repeatmasker.org/) in a WU-BLASTX search against the transposable element protein database to further identify repeat-related proteins. The overlapping transposable elements belonging to the same repeat class were collated and combined according to the coordination in the genome. In addition, we annotated the tandem repeats by using the software Tandem Repeats Finder^80^ (http://tandem.bu.edu/trf/trf.html).

The full-length *Tam* sequences were retrieved from the NCBI nucleotide database and used to query the genome with BLASTN (v.4x10, -minIdentity=70). Hits with more than 20% query alignment coverage were retrieved with genomic coordinates. These sequences were then subjected to group analysis with blastclust (-S100-L0.99) integrated within NCBI BLAST (blast-2.2.25). Candidate recent active clusters were further examined by self-BLASTN of member sequences within these clusters and were considered supported where 100% query coverage and 100% identity (without mismatch or indels) were reported. For *Tam1*, *Tam2*, *Tam4* and *Tam11*, there was only one full-length *Tam* sequence detected. For *Tam3*, BLAST searches using each of five different full-length *Tam3* sequence accessions (AB012941, 3,698 bp; AB005454, 3577 bp; AB038403, 3488 bp; AB038404, 3601 bp; AB038404, 3,611 bp) produced similar results to the initial cloned *Tam3* (X55078, 3,629 bp) and confirmed that the *A. majus* genome sequenced in this study did not have a recent active cluster.

### Gene family and synteny

To identify gene family groups, we analysed protein-coding genes from nine plant species, *A. majus* (this study), *S. tuberosum* (ftp://ftp.ncbi.nlm.nih.gov/genomes/Solanum_tuberosum/)^81^, *P. axillaris* (ftp://ftp.solgenomics.net/genomes/Petunia_axillaris/)^82^, *P. inflata* (ftp://ftp.solgenomics.net/genomes/Petunia_inflata/)^82^, *S. lycopersicum* (http://www.ncbi.nlm.nih.gov/genome/annotation_euk/Solanum_lycopersicum/101/)^83^, *A. thaliana* (https://www.arabidopsis.org/, TAIR10)^84^, *C. papaya* (http://www.plantgdb.org/CpGDB/, v1. 81)^85^, *P. mume* (https://www.rosaceae.org/, v2.0.a1)^86^, *V. vinifera* (http://www.genoscope.cns.fr/externe/Download/Projets/Projet_ML/data/12X/)^87^, *O*. *sativa* (http://rice.plantbiology.msu.edu/)^88^ and *A. trichopod* (http://www.amborella.org/)^89^. We performed an all-against-all comparison using BLASTP^57^ with an *E* value cut-off of 1 × 10^-5^, and the OrthoMCL method^90^ was used to cluster the BLASTP results into paralogous and orthologous clusters.

In total, 2,115 single-copy gene families were used to reconstruct the phylogenetic tree. First, the proteins of single-copy gene families were aligned by MUSCLE^91^. Following alignment, the protein was reverse-transcribed into the coding sequence and four-fold degenerate sites were extracted from each alignment and concatenated to create one super gene for each species. We used jModelTest to select the best model (http://darwin.uvigo.es)^92^. PhyML^93^ was used to construct the phylogenetic tree using the JTT+I+GAMMA model and 1,000 bootstrap replicates. We used a similar method to PGDD (http://chibba.agtec.uga.edu/duplication/) to identify WGD events within the *A. majus* genome. Proteins were aligned to each other with BLASTP and a filter threshold of 1 × 10^-5^ was used to identify homologous proteins. MCScanX^75^ with default parameters (http://chibba.pgml.uga.edu/mcscan2/) was used to find collinear blocks, each containing at least five collinear gene pairs. The Ks value was calculated with the PAML^94^ yn00 NG model (http://abacus.gene.ucl.ac.uk/software/paml.html). Intragenome dot plot comparison of *A. majus* was carried out using the SynMap tool from the online CoGe portal^95^ (http://genomevolution.org/CoGe/). GEvo microsyntenic analysis of each collinear block was performed using SynMap and SynFind also in the CoGe portal. The divergence times of *C. papaya***–***A. thaliana* (~55.1–90.6 Ma) and dicot–monocot (~123.9–228.5 Ma) were used for calibration.

### Transcriptome analysis

Total RNA was isolated from the leaf, pistil, pollen, root, stamen and stem. For tissues with large biomass (leaf, root and stem), each tissue mixture was obtained from three plants. For the rest of the tissues (pistil, pollen and stamen), each tissue mixture was sampled from at least 10 plants. All plants used in RNA-seq were growth at the environment as that used for genome sequencing and were confirmed with consistent growth. RNA sequencing libraries (300–500 bp fragments) were constructed using the mRNA-Seq Prep Kit (Illumina). Then, we sequenced all libraries using Illumina HiSeq 2000 (2 × 100 bp). FastQC^96^ qualified reads ware mapped to the genome guided by the final gene model using hisat2 (http://www.bioinformatics.babraham.ac.uk/projects/fastqc) and the expression level for each gene was calculated by Stringtie^97^.

### Construction of BAC library

High-molecular-weight DNA of over 2 Mb from *Antirrhinum* (*A. majus* JI7 line) was extracted from leaf nuclei according to Liu and Whitter^98^ and partially digested with *Hin*dIII. BAC vector preparation, ligation and transformation of *TranformMaxTM EP1300TM Escherichia coli* (EPICENTRE Biotechnologies) by electroporation followed the described method^99^. BAC DNA was digested with *Hin*dIII and sized-fractioned with a field inversion agarose gel electrophoresis to estimate the insert length. A total of 114,816 clones were selected and stored in 384-well plates.

### FISH

Immature 1.5–3.0 mm *Antirrhinum* flower buds were harvested and fixed in Carnoy’s solution (ethanol:glacial acetic acid, 3:1) and stored at –20 °C. BAC clones were isolated and labelled with digoxigenin-16-dUTP or biotin-11-dUTP by nick translation. FISH was performed on the pachytene chromosomes as described^98^. Chromosomes were counterstained with 4’6-diamindino-phenylindole (DAPI) in an antifade solution. Chromosomes and FISH signal images were captured with an Olympus BX53 fluorescence microscope conjunct with a micro charge-coupled device camera. Three experiments were performed^100^.

### Evolutionary analysis of TCP family genes

Syntenic block identification and Ks analyses were carried out using MCscanX^75^ and the PAML^94^ yn00 NG model, respectively. MEGA7^101^ was used for the multiple alignment and phylogentic tree construction. Expression pattern was carried out with MeV. TF family annotation was carried out using the website of plantTFDB (http://planttfdb.cbi.pku.edu.cn/prediction.php).

The annotations and sequences of *Aquilegia coerulea*^102^ were downloaded from the website https://img.jgi.dofor exampleov/. *Phalaenopsis equestris*^103^ was downloaded from http://orchidbase.itps.ncku.edu.tw/.

*Petunia axillaris* and *Petunia inflata* were downloaded from https://solgenomics.net/organism/Petunia_axillaris/genome and https://solgenomics.net/organism/Petunia_inflata/genome, respectively.

The functional known protein members in TCP families were downloaded from the original experimental papers and used as marker proteins for TCP subfamily identification. Two putative TCP family genes (*Am03g34120* with partial TCP domains and *Am01g42140* with two tandem TCP domains) were excluded from phylogenetic analyses. Syntenic block and Ks analysis detected three gene pairs derived from WGD. *Am08g22680*/*Am06g32830* (Ks = 0.99) and *Am08g20570*/*Am06g35450* (Ks = 0.76) were located in a large syntenic block with 79 homologous gene pairs (median Ks = 0.85), while *Am08g18340*/*Am06g39840* (Ks = 0.58) were located in a block with 11 homologous gene pairs (median Ks = 1.01).

Known MYB family TFs involved in zygomorphic flower control, *DIV* (Q8S9H7), *DIVL*(AAL78742), *RAD*(Q58FS3), *DRIF-1*(AGL11918) and *DRIF-2* (AGL11919), were BlastP searched against the proteome. The protein sequences for the top two best hits were retrieved and confirmed by InterPro analysis. Retained duplicated copies derived from the WGD event were confirmed by both syntenic block and Ks analysis. Expression and function analysis identified *Lotus japonicus* *CYC* genes *LjCYC1* (DQ202475), *LjCYC2* (DQ202476), *LjCYC3* (DQ202477) and *LjCYC5* (DQ202478) that were used to BlastP^57^ search the *L japonicas* proteome. All three *CYC* genes could be detected with ≥98% amino acid identity except for *LjCYC2*, which was not detected with relaxed alignment criteria nor by Tblastn search *of* the *L. japonicas* genome (downloaded from http://chibba.agtec.uga.edu/duplication v.2.5), strongly indicating that the *LjCYC2* gene was absent from the current *L. japonicas* assembly.

### Genome-wide search for *S*-gene candidates in *Antirrhinum* and related plant species

Except for the genome data set of *Antirrhinum*, the recently published or revised versions of the other 14 genome data sets were downloaded from their public websites (Supplementary Data Set 14). Published SLF protein sequences of *Antirrhinum* were separately used to establish the group-specific SLF profiles of Plantaginaceae.

Alignments were verified manually, and a consensus sequence was created for each of the motifs of interest with the help of the Weblogo software package^104^. This alignment was used to generate an HMM model using the program hmmbuild from the HMMER program suite40. Using hmmsearch, the HMM model was applied in a search against the most recent protein annotations from each plant species. To confirm the presence of both F-box and Kelch/FBA1/FBA3 domains in the obtained sequences (*E* < 10^–30^), we further compared the results from hmmsearch and the Pfam databases with the hmmpfam package. Our domains of interest were annotated in Pfam as PF00646 (F-box), PF04300 (FBA1), PF08268 (FBA3) and PF01344 (Kelch domain 1).

### Reporting Summary

Further information on research design is available in the Nature Research Reporting Summary linked to this article.

## Supplementary information

Supplementary Information Supplementary Figures 1–10 and Supplementary Tables 1–14.

Reporting Summary

Supplementary Data Supplementary Data Set 1–14.

## Data Availability

Genome assembly data have been deposited at NCBI BioProject ID under accession codes PRJNA227267. The raw sequence data reported in this paper have been deposited in the Genome Sequence Archive^105^ in the BIG Data Center^106^, Beijing Institute of Genomics (BIG), Chinese Academy of Sciences, under accession numbers PRJCA000223 and PRJCA001050 that are publicly accessible at http://bigd.big.ac.cn/gsa. We built the *Antirrhinum* genome website at http://bioinfo.sibs.ac.cn/Am, providing a portal to genome browser, Blast, data download and gene expression functions. All data that support the findings of this study are also available from the corresponding authors upon request.
