# Supplementary material for: Genome structure and evolution of Antirrhinum majus L
Source: Nat Plants. 2019 Jan 28;5(2):174–83. doi: 10.1038/s41477-018-0349-9 (PMC6784882; doi:10.1038/s41477-018-0349-9)
Supplement: Supplementary file 1 — Supplementary Figures 1–10 and Supplementary Tables 1–14. [file 41477_2018_349_MOESM1_ESM.pdf]

nature plants

SUPPLEMENTARY INFORMATION

https://doi.org/10.1038/s41477-018-0349-9

In the format provided by the authors and unedited.

Genome structure and evolution of *Antirrhinum majus* L

Miaomiao Li[1,2,8], Dongfen Zhang[1,8], Qiang Gao[3,8], Yingfeng Luo[4,8], Hui Zhang[1,2,8], Bin Ma[3,8], Chunhai Chen[5,8], Annabel Whibley[6], Yu'e Zhang[1], Yinghao Cao[3], Qun Li[1], Han Guo[1,2], Junhui Li[1,2], Yanzhai Song[1,2], Yue Zhang[1,2], Lucy Copsey[6], Yan Li[3], Xiuxiu Li[2,3], Ming Qi[3], Jiawei Wang[7], Yan Chen[5], Dan Wang[5], Jinyang Zhao[5], Guocheng Liu[5], Bin Wu[5], Lili Yu[5], Chunyan Xu[5], Jiang Li[5], Shancen Zhao[5], Yijing Zhang[7], Songnian Hu[2,4], Chengzhi Liang[2,3*], Ye Yin[5*], Enrico Coen[6*] and Yongbiao Xue[1,2,4*]

[1]State Key Laboratory of Plant Cell and Chromosome Engineering and National Center of Plant Gene Research, Institute of Genetics and Developmental Biology, Chinese Academy of Sciences, Beijing, China. [2]University of Chinese Academy of Sciences, Beijing, China. [3]State Key Laboratory of Plant Genomics, Institute of Genetics and Developmental Biology, Chinese Academy of Sciences, Beijing, China. [4]Beijing Institute of Genomics, Chinese Academy of Sciences, Beijing, China. [5]BGI-Shenzhen, Shenzhen, China. [6]John Innes Centre, Norwich, UK. [7]National Laboratory of Plant Molecular Genetics, CAS Center for Excellence in Molecular Plant Sciences, Institute of Plant Physiology and Ecology, Shanghai Institutes for Biological Sciences, Chinese Academy of Sciences, Shanghai, China. [8]These authors contributed equally: Miaomiao Li, Dongfen Zhang, Qiang Gao, Yingfeng Luo, Hui Zhang, Bin Ma, Chunhai Chen *e-mail: cliang@genetics.ac.cn; yinye@genomics.cn; enrico.coen@jic.ac.uk; ybxue@genetics.ac.cn

Supplementary Figures

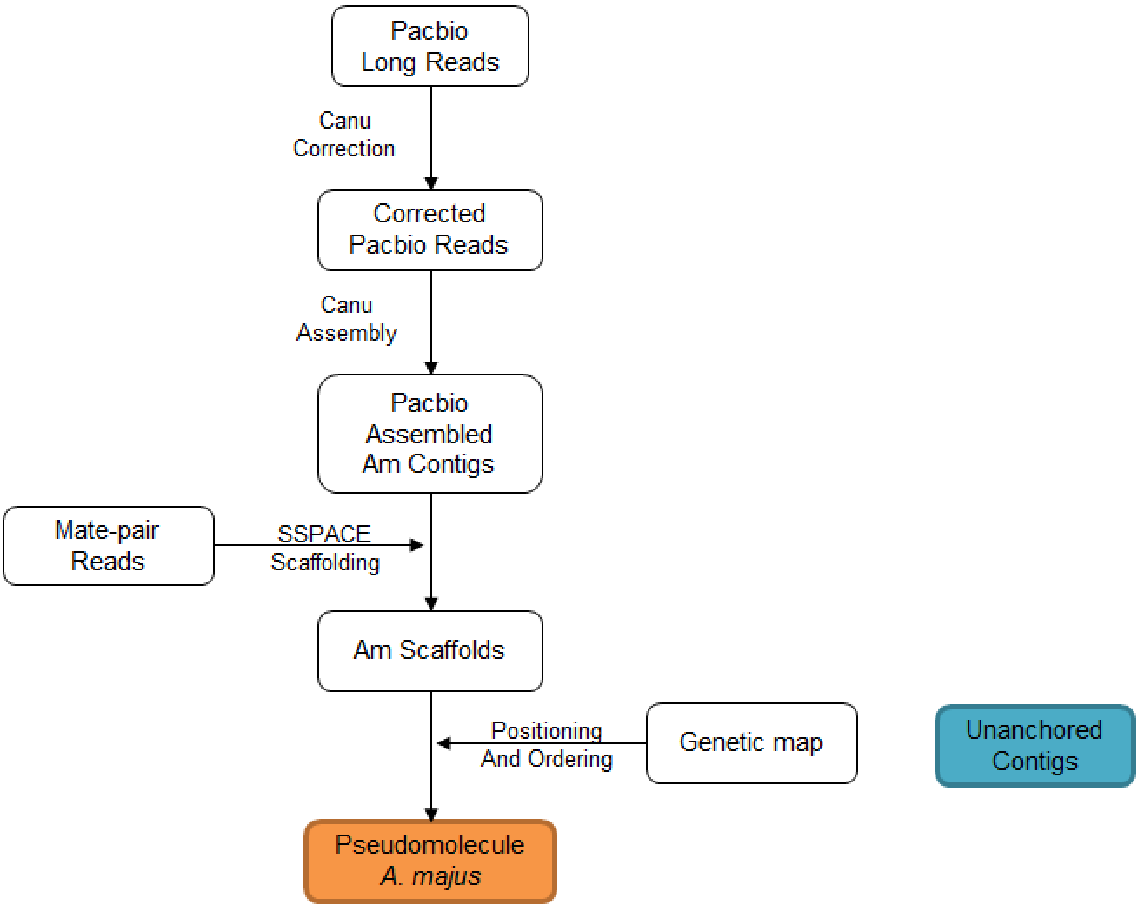

Supplementary Figure 1. Flow chart showing the genome assembly process. The reads from Illumina and PacBio sequencing were assembled to scaffolds, which were anchored to different linkage groups by genetic mapping.

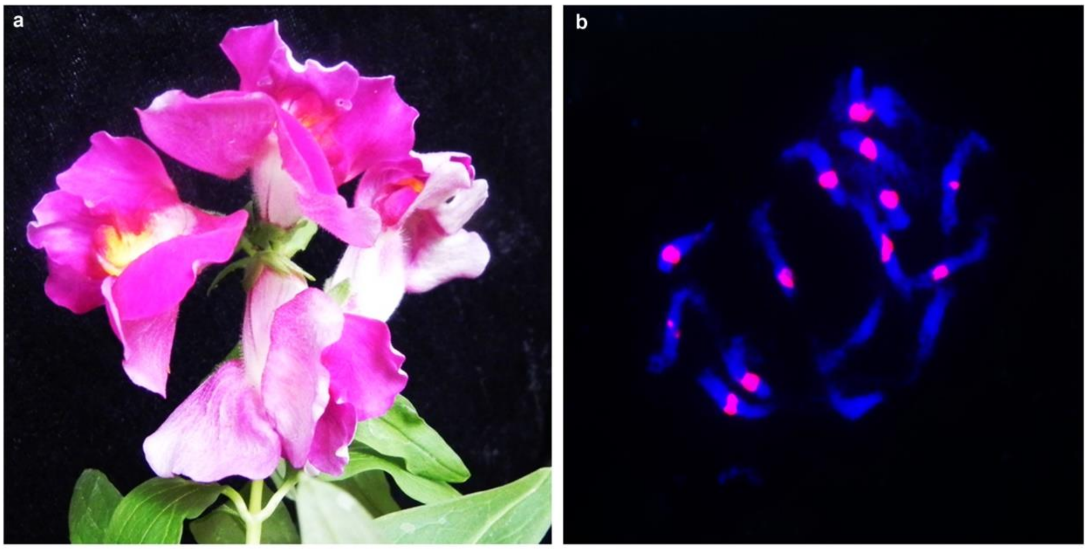

Supplementary Fig. 2. Mature flowers of and chromosome karyotype of *Antirrhinum*.

a, Mature flowers of *A. majus* JI7.**b,** Fluorescence in situ hybridization of *Antirrhinum* chromosomes (2n=16) at mitotic metaphase with the *centA1* probe (red) labeled with biotin and detected with FITC. The chromosomes were stained by 4',6-diamidino-phenylindole (DAPI). The experiments were repeated three times independently with similar results.

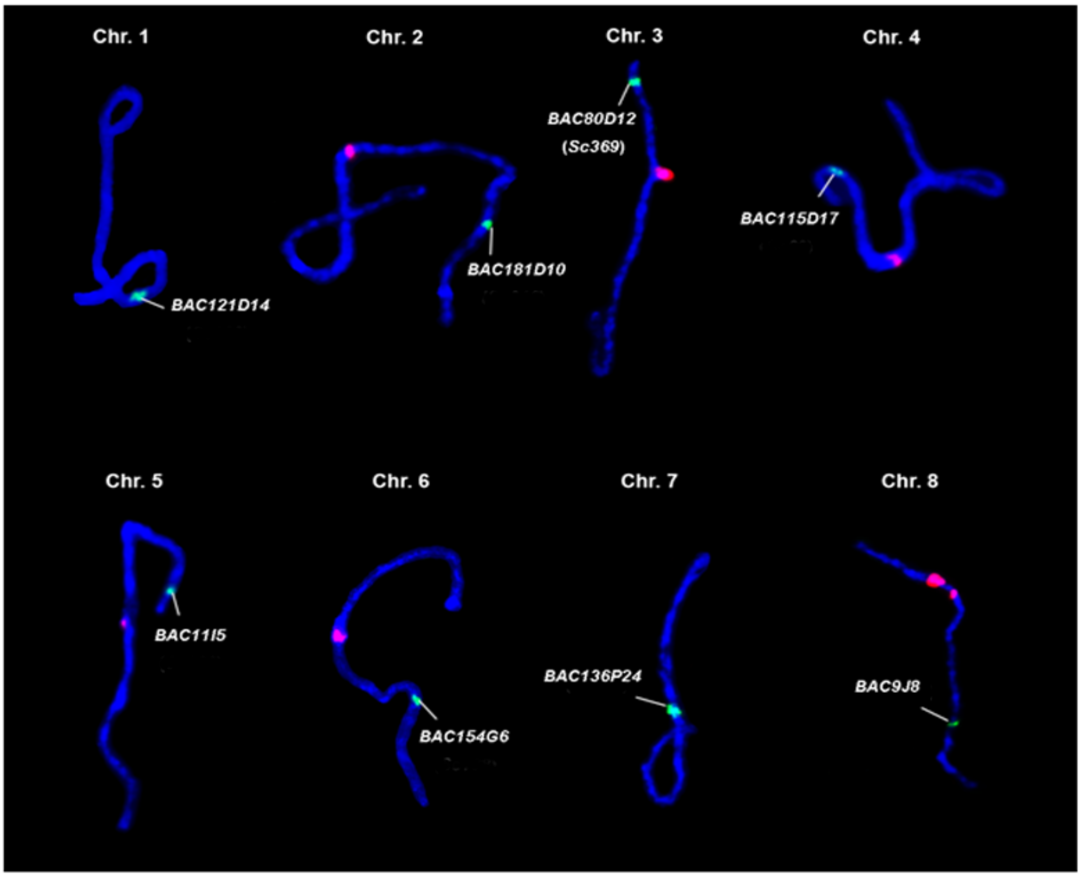

Supplementary Figure 3. The link of Eight *Antirrhinum* linkage groups to chromosomes by FISH.

Eight BAC clones from an *Antirrhinum* BAC library representing the eight linkage groups were used for FISH to confirm the chromosomal locations of the assembled genome sequence. The chromosomes are shown in pseudo blue color by 4’, 6-diamidinophenylindole (DAPI) staining. The positions of BAC clones (green signals) representing each scaffold as well as *Antirrhinum* centromere *CentA1* (red signals) on the chromosomes. The FISH experiments were repeated three times independently with similar results.

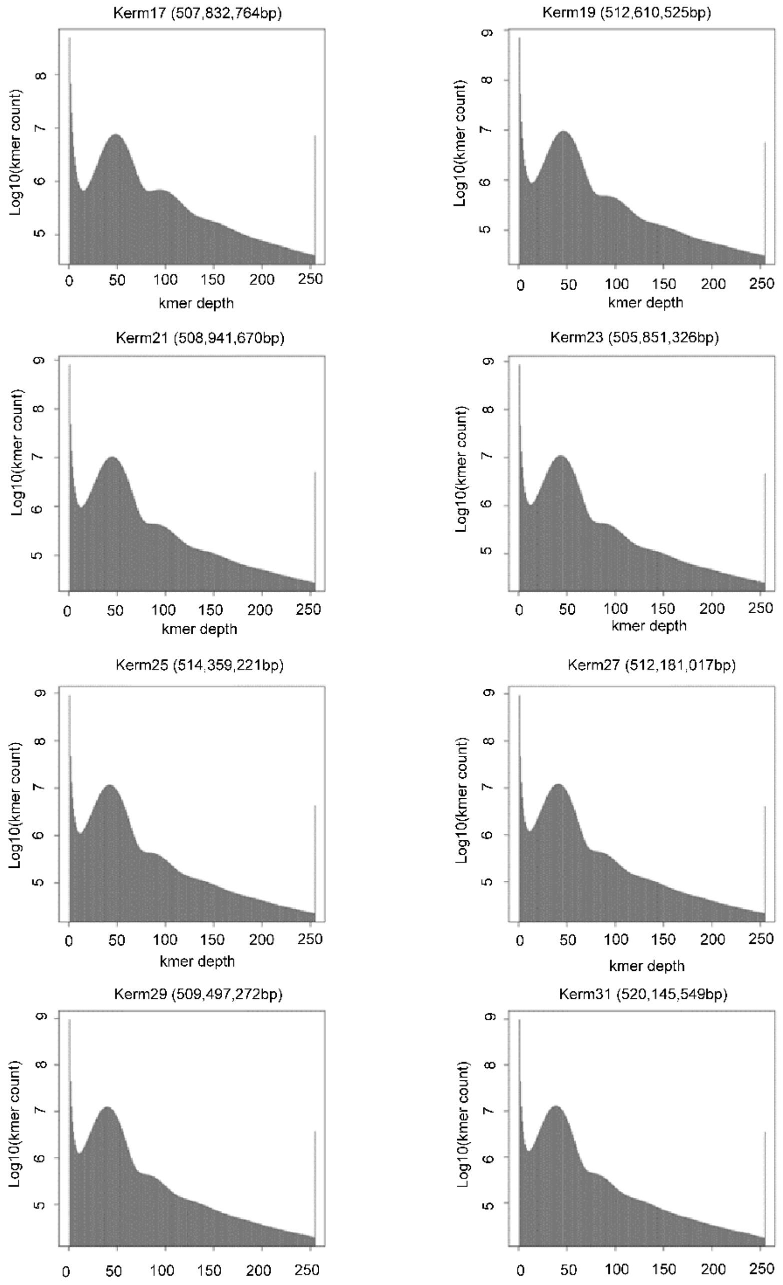

Supplementary Fig. 4 Distribution of 17-mer, 19-mer, 21-mer, 23-mer, 25-mer, 27-mer, 29-mer, 31-mer in raw sequence data of *A. majus* JI7. The estimated genome size is shown in the brackets for each kmer. The x-axis and y-axis separately indicate the kmer length and number, respectively.

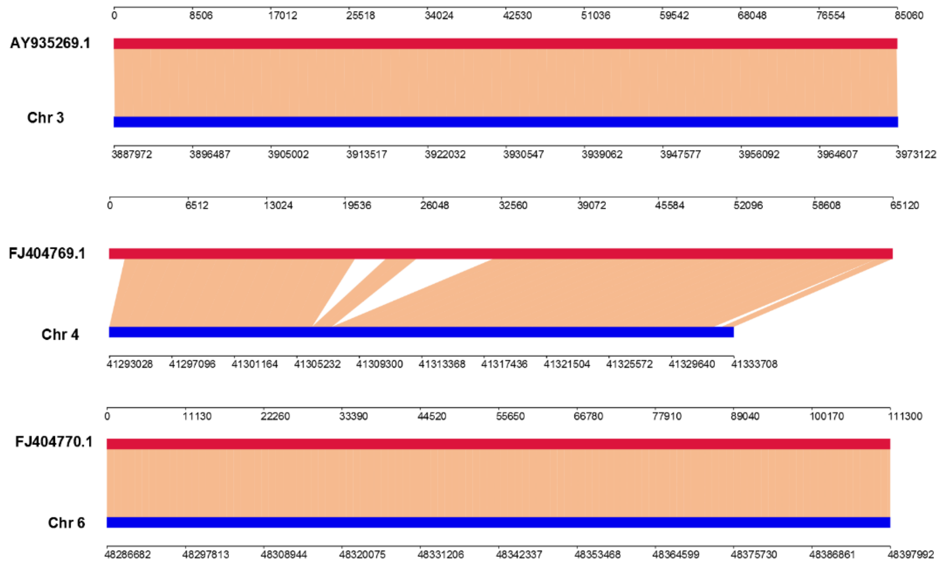

Supplementary Fig. 5 Genome quality evaluated using three sequenced BACs as references and NGS data mapping results. The reference BAC sequences (GeneBank accession numbers: AY935269.1, FJ404769.1 and FJ404770.1) were aligned to the assembled *Antirrhinum* genome using BWA. The identity of AY935269.1, FJ404769.1 and FJ404770.1 are 99.98%, 98.15% and 99.99% separately. Blue lines show the assembled *Antirrhinum* genome sequence, red BAC sequences and thick yellow blocks the synteny between BAC clones and the assembled *Antirrhinum* genome.

a

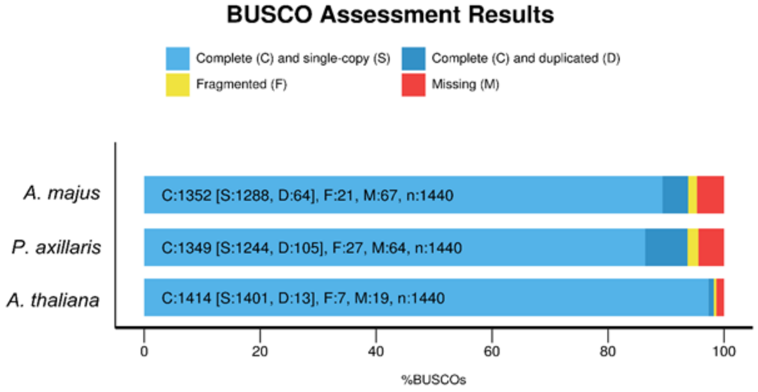

b

Types	Genome	proteins
Total embryophyta BUSCO groups searched	1,440	1,440
Complete BUSCOs	1,352	1,345
Complete and single-copy BUSCOs	1,288	1,098
Complete and duplicated BUSCOs	64	247
Fragmented BUSCOs	21	33
Missing BUSCOs	67	62

Supplementary Fig.6 BUSCO analysis of genome quality and completeness. a, Complete and single-copy (blue) or duplicated genes (dark blue) are shown in three species *A. majus*, *P. axillaris* and *A. thaliana*. Gene fragmented and missing gene are shown in yellow and red, respectively. **b,** The assessments using benchmarking universal single-copy orthologs analysis on the genome and the protein set.

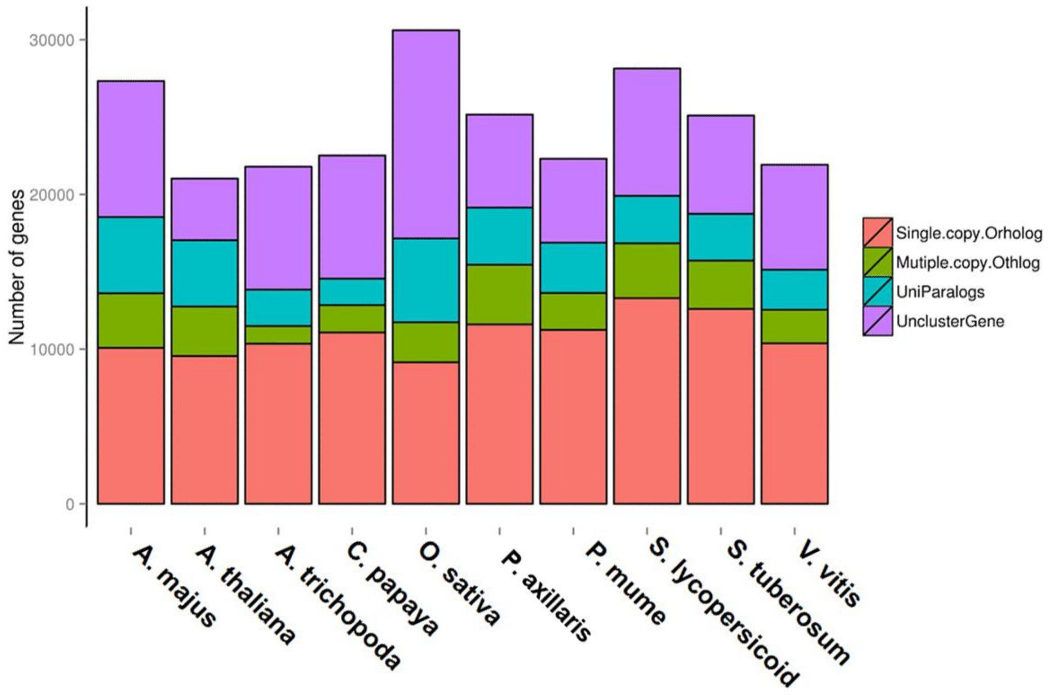

Supplementary Fig. 7 Comparisons of ortholog protein families among ten plant species. *A. majus*, *A. thaliana*, *A. trichopoda*, *C. papaya*, *O. sativa*, *P. axillaris*, *P. mume*, *S. lycopersicum*, *S. tuberosum* and *V. vitis* were used for analysis. Species names are shown on the x-axis and the gene numbers of orthologous or paralogous sequences in the genome on the y-axis.

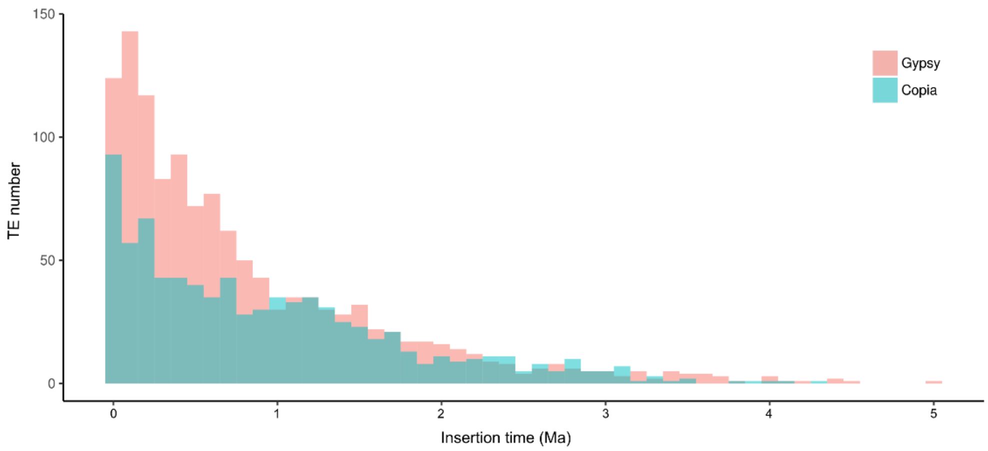

Supplementary Fig. 8 Calculation of insertion times of LTR retrotransposons of *Gypsy* and *Copia* in *A. majus*. The x-axis and y-axis show insertion time and TE number, respectively.

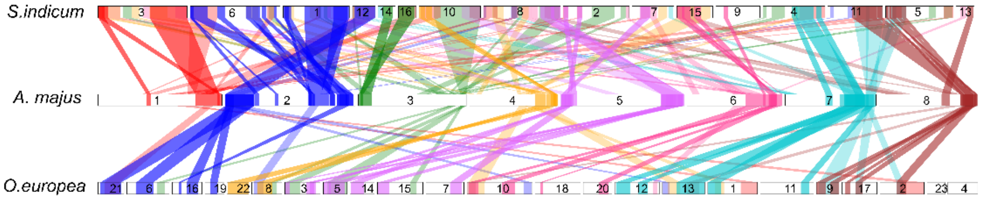

Supplementary Fig. 9 Synteny blocks among chromosomes of *A. majus*, *S. indicum* and *O. europea.*

The numbers represent individual chromosomes. The selected syntenic gene numbers are more than fifty in each block.

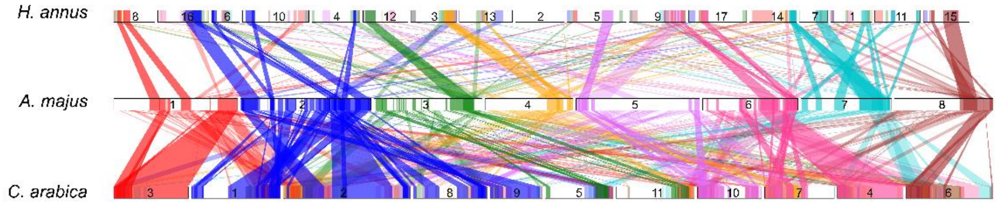

Supplementary Fig. 10 Synteny blocks among chromosomes of *A. majus*, *H. annus* and *C. arabica*. The numbers represent individual chromosomes. The selected syntenic gene numbers are more than fifteen in each block between *H. annus* and *A. majus*; more than fifty in each block between *C. arabica* and *A. majus*, respectively.

Supplementary Tables

Supplementary Table 1 Estimate of *A. majus* JI7 genome size.

cleandataMC	N	L	K	B	D	G
TotalData	297,302,586	100	17	597,444,555	48	507,832,764
			19	798,727,921	46	512,610,525
			21	881,831,719	45	508,941,670
			23	932,143,354	44	505,851,326
			25	970,909,261	42	514,859,221
			27	1,000,969,655	41	512,181,017
			29	1,025,895,294	40	509,497,272
			31	1,045,650,168	38	520,145,549

Genome size was estimated by K-mer distributions. Abbreviations: total sequence read number (N), the average length of sequence reads (L), kmer length defined as 17 -31 bp here (K), total number of low frequency K-mer (B), the overall depth estimated from K-mer distribution (D) and GenomeSize (G)

Supplementary Table 2 Global statistics of Solexa sequencing data for *A.majus*.

Pair-end libraries	Insert size	Reads length	Raw data			Clean data		
			Total data (Gb)	Sequence coverage (X)[a]	Physical coverage (X)[b]	Total data (Gb)	Sequence coverage (X)	Physical coverage (X)
Solexa	170bp	100bp	25.19	39.98	33.98	22.13	35.13	29.86
Reads	500bp	100bp	14.99	23.79	59.48	11.98	19.02	47.55
	800bp	100bp	9.77	15.51	62.04	7.11	11.29	45.16
	2,000bp	49bp	8.51	13.51	270.2	6.2	9.84	196.8
	5,000bp	49bp	13.95	22.14	1107	2.65	4.21	210.5
	10,000bp	49bp	5.77	9.16	916	1.62	2.57	257
	20,000bp	49bp	12.67	20.11	4022	1.12	1.78	356
**Total**	--	--	90.85	144.2	6470.7	52.81	83.84	1142.87

[a] Sequence coverage is the average number of times a base is read.

[b] Physical coverage is the average number of times a base is read or spanned by mate-paired reads.

The raw sequence data were deposited in the Genome Sequence Archive in BIG Data Center, under accession numbers SAMC005769, submission ID subSAM006464 which are publicly accessible at http://bigd.big.ac.cn/gsa.

Supplementary Table 3 PacBio sequencing data for *A. majus*.

SMRT(#)	30SMRTs		40SMRTs	
**Metrics**	**Pre-Filter**	**Post-Filter**	**Pre-Filter**	**Post-Filter**
Polymerase Read Bases	27,589,242,675	25,894,294,144	35,932,570,581	33,843,699,345
Polymerase Reads	4,508,760	2,234,998	6,011,680	2,973,433
Polymerase Read N50	15,451	15,678	15,247	15,463
Polymerase Read Length	6,119	11,585	5,977	11,382
Polymerase Read Quality	0.434	0.828	0.433	0.829

The summary of PacBio reads containing size, length and quality is given.

Supplemental Table 4 Summary of *A. majus* JI7 genome assembly.

	Contig (bp)	Scaffold (bp)
n (number)	1,645	620
n: N50	223	62
Min	3,581	4,661
N90	339,595	1,186,578
N80	339,595 b	1,186,578
N50	733,198	2,619,411
N20	1,263,020	4,803,629
Max length	3,740,391	9,899,914
Total Length	510,195,251	511,700,000

The number and size of both contigs and scaffolds including in the genome assembly are given.

Supplemental Table 5 The NGS data mapping result.

	Depth	Coverage	Mapping Ratio	Properly Paired Mapping
100917_I145_FC801W4ABXX_L1_ANTwnrDAWDIAAPEI-7	19.71	98.7	99.60%	98.21%
101018_I117_FC807FGABXX_L7_ANTwnrDAWDBAAPEI-7	11.53	97.94	99.33%	96.89%
100917_I145_FC801W4ABXX_L6_ANTwnrDAWDMBAPEI-2	10.98	96.6	99.72%	98.69%

The average mapping ratio was 99.55%.

Supplemental Table 6 Summary of *A. majus* JI7 pseudomolecules.

Chr	Length (bp)	Gene number	Genetics Map (cM)	Maker Number	Contigs Number
Chr1	71721304	6535	114.20	312	168
Chr2	75456924	5605	123.57	297	174
Chr3	62720994	4460	94.70	323	153
Chr4	50908493	4426	78.09	190	108
Chr5	71569243	4693	136.03	314	162
Chr6	55037871	4030	122.94	203	122
Chr7	52493632	3717	124.16	203	117
Chr8	57078755	3768	100.03	190	123
Total	496,987,216	37234	893.74	2,032	1,127

Average recombination rate is 1.798 cM /Mb.

Supplementary Table 7 Gene numbers and features of *A. majus* JI7.

Gene numbers and features	
Gene number	37714
Max gene length	76453
Min gene length	200
Gene length*	2,266/3,166
mRNA length*	1,190/1,404
Max cds length	15258
CDS length*	846/1,036
Protein length*	281/344
Exon length*	163/314
Intron length*	209/488
5' UTR length*	126/209
3' UTR length*	221/292
Exon number*	3.0/4.5
Intron number*	2.0/3.5
Transcript number**	1.0/1.4

* Means the median/average length in base pair (bp).

** Means the median/average transcript number per gene.

Supplementary Table 8 Functional annotation of the predicted genes in the assembly.

	Number	Percent (%)
InterPro	29732	78.83
GO	21516	57.05
Pathway	5184	13.74
Pfam	28476	75.50
Sequence Similarity (Arabidopsis)	16271	43.14
Annotated	33608	89.11
Unannotated	4106	10.88
Total	37714	100

Different databases were used to annotate genes in *A. majus* JI7 genome, along with their corresponding percentage of the total genes identified.

Supplementary Table 9 Functional annotation of the predicted genes in the assembly.

Type	Copy (w)	Average length (bp)	Total length (bp)	Percentage in genome (1e^{-4})
RNA	800	165	131,929	2.58
RNA	981	75	73,863	1.44
RNA	10	4223.8	42,238	0.82
18S	45	2403	108,114	2.11
28S	24	3064	73,534	1.44
5.8S	19	163	3,097	0.06
5S	29	121	3,515	0.06
snRNA	622	388	77,472	1.51
CD-box	352	116	40,728	0.79
HACA-box	59	136	8,016	0.15
Splicing	211	136	28,728	0.56

The number, length and percentage of different type of non-coding RNA genes are shown.

Supplementary Table 10 Summary of repeat DNA in *A. majus* JI7.

	Length (bp)	Percentage of genome (%)
Class I: Retrotransposon	182,874,393	35.84
LTR-Retrotransposon	163,759,196	32.09
LTR/Gypsy	71,314,590	13.97
LTR/Copia	79,489,591	15.58
Other	12,955,015	2.53
Non-LTR Retrotransposon	19,115,197	3.74
SINE	2,928,943	0.57
LINE	16,186,254	3.17
Class II: DNA Transposon	41,170,089	8.06
EnSpm/CACTA	27,898,593	5.46
hAT	4,851,532	0.95
Harbinger	785,115	0.15
Tc1/Mariner	1,482,313	0.29
MuDR	4,240,104	0.83
Helitron	876,495	0.17
Other	1,035,937	0.20
Tandem repeat	13,034,642	2.55
Low Complexity	1,323,673	0.25
Unclassified	29,943,821	5.86
Total content	268,346,618	52.5

Genome composition by repeat DNA, the type, length and percentage are given.

Supplementary Table 11 The summary of *Tam* transposon in *A. majus* JI7.

Tam type	Accession	Hits*	Best hit query coverage (%)	Best hit Identity (%)	Active cluster name/Hit copies
Tam1	X57297	2	100%	99.69%	0/0
Tam2	X06266	3	41.64%	94.76%	*Tam2*-cluster1/2
Tam3	X55078	66	100%	99.86%	0/0
Tam4	X59057	16	100%	99.07%	*Tam4*-cluster1/14
Tam11	AY077455	8	100%	99.28%	*Tam11*-cluster11/6

*The number of detected Tam sequences for each type.

Supplementary Table 12 Gene Set Enrichment Analysis (GSEA) of *A. majus* JI7.

GO ID	Description	GO class	Gene frequency*
GO:0000148	1,3-beta-D-glucan synthase complex	cellular_component	12/27
GO:0000159	protein phosphatase type 2A complex	cellular_component	11/21
GO:0003735	structural constituent of ribosome	molecular_function	70/406
GO:0003774	motor activity	molecular_function	12/27
GO:0003824	catalytic activity	molecular_function	289/1626
GO:0003830	beta-1,4-mannosylglycoprotein 4-beta-N-acetylglucosaminyltransferase activity	molecular_function	6/10
GO:0003849	3-deoxy-7-phosphoheptulonate synthase activity	molecular_function	5/7
GO:0003924	GTPase activity	molecular_function	42/211
GO:0003995	acyl-CoA dehydrogenase activity	molecular_function	15/37
GO:0004252	serine-type endopeptidase activity	molecular_function	42/209
GO:0004553	hydrolase activity, hydrolyzing O-glycosyl compounds	molecular_function	92/490
GO:0004672	protein kinase activity	molecular_function	402/1662
GO:0004970	ionotropic glutamate receptor activity	molecular_function	10/28
GO:0005089	Rho guanyl-nucleotide exchange factor activity	molecular_function	11/17
GO:0005215	transporter activity	molecular_function	116/384
GO:0005216	ion channel activity	molecular_function	17/47
GO:0005315	inorganic phosphate transmembrane transporter activity	molecular_function	18/19
GO:0005507	copper ion binding	molecular_function	25/101
GO:0005524	ATP binding	molecular_function	156/719
GO:0005886	plasma membrane	cellular_component	9/23
GO:0006631	fatty acid metabolic process	biological_process	8/11
GO:0006855	drug transmembrane transport	biological_process	44/129
GO:0008519	ammonium transmembrane transporter activity	molecular_function	11/15
GO:0009522	photosystem I	cellular_component	12/24
GO:0015079	potassium ion transmembrane transporter activity	molecular_function	20/33
GO:0016614	oxidoreductase activity, acting on CH-OH group of donors	molecular_function	7/14
GO:0046373	L-arabinose metabolic process	biological_process	5/7
GO:0055085	transmembrane transport	biological_process	31/102

*Expansion-family genes/all genes in each GO category

Supplementary Table 13 Transcription factor family member numbers among *Antirrhinum*, *Arabidopsis*, *Solanum*, *Vitis*, *Sesamumindicum* and *Oeuropaea.*

**Gene**	***Antirrhinum***	***Arabidopsis***	***Solanum***	***Vitis***	***Sesamumindicum***	***Oeuropaea***
AP2/ERF-AP2	19	13	22	15	34	25
AP2/ERF-ERF	170	124	140	81	135	227
AP2/ERF-RAV	3	4	3	1	10	4
C2H2	139	106	116	76	149	198
GRAS	88	34	54	43	84	88
TCP	32	24	36	15	35	55
Trihelix	43	26	25	23	49	42

The name and number of transcription families are given across six species.

Supplementary Table 14 The evolutionary analysis of RAD/DIV module in *A. majus* JI7.

Gene A name	Gene B name curated	Gene A ID	Gene B ID	Syntenic Block	Best hit	Ks
DIV	DIVL	Am06g28300	Am02g27700	None	Yes	1.01
DIV	DIVL-2	Am06g28300	Am08g35450	Yes	No	2.81
DIV	DIVL-3	Am06g28300	Am07g36510	yes	No	5.24
RAD	RADL-1	Am08g05060	Am05g44710	None	Yes	1.63
RAD	RADL-2	Am08g05060	Am01g45570	yes	No	71.27
RAD	RADL-3	Am08g05060	Am04g25700	yes	No	67.69
DRIF-1	DRIF-1L-1	Am02g19380	Am01g35780	Yes	Yes	0.81
DRIF-1	DRIF-1L-2	Am02g19380	Am01g08360	Yes	No	9.31
DRIF-2	DRIF-2L	Am01g19370	Am07g07870	None	Yes	0.79

Syntenic block and *Ks* analysis were used to evaluate the gene pairs (A and B). “None” means two genes are not located in syntenic block. “No” means two genes are not identified as reciprocal best hits using all-versus-all BLASTP analysis.
