# Supplementary material for: Genome structure and evolution of Antirrhinum majus L
Source: Nat Plants. 2019 Jan 28;5(2):174–83. doi: 10.1038/s41477-018-0349-9 (PMC6784882; doi:10.1038/s41477-018-0349-9)
Supplement: Supplementary file 2 — Reporting Summary [file 41477_2018_349_MOESM2_ESM.pdf]

nature research

Corresponding author(s): Yongbiao Xue

Reporting Summary

Nature Research wishes to improve the reproducibility of the work that we publish. This form provides structure for consistency and transparency in reporting. For further information on Nature Research policies, see Authors & Referees and the Editorial Policy Checklist.

Statistical parameters

When statistical analyses are reported, confirm that the following items are present in the relevant location (e.g. figure legend, table legend, main text, or Methods section).

n/a	Confirmed	
☐	☒	The exact sample size (*n*) for each experimental group/condition, given as a discrete number and unit of measurement
☐	☒	An indication of whether measurements were taken from distinct samples or whether the same sample was measured repeatedly
☐	☒	The statistical test(s) used AND whether they are one- or two-sided<br>*Only common tests should be described solely by name; describe more complex techniques in the Methods section.*
☒	☐	A description of all covariates tested
☐	☒	A description of any assumptions or corrections, such as tests of normality and adjustment for multiple comparisons
☐	☒	A full description of the statistics including central tendency (e.g. means) or other basic estimates (e.g. regression coefficient) AND variation (e.g. standard deviation) or associated estimates of uncertainty (e.g. confidence intervals)
☐	☒	For null hypothesis testing, the test statistic (e.g. *F*, *t*, *r*) with confidence intervals, effect sizes, degrees of freedom and *P* value noted<br>*Give P values as exact values whenever suitable.*
☒	☐	For Bayesian analysis, information on the choice of priors and Markov chain Monte Carlo settings
☒	☐	For hierarchical and complex designs, identification of the appropriate level for tests and full reporting of outcomes
☒	☐	Estimates of effect sizes (e.g. Cohen's *d*, Pearson's *r*), indicating how they were calculated
☐	☒	Clearly defined error bars<br>*State explicitly what error bars represent (e.g. SD, SE, CI)*

Our web collection on statistics for biologists may be useful.

Software and code

Policy information about availability of computer code

Data collection

we constructed a total of 2×100 paired-end sequencing libraries with insert sizes from 170 bp to 20 kb for standard WGS sequencing using Illumina HiSeq 2000./2.Using the Pacific Biosciences (PacBio) platform for single-molecule, real time (SMRT) sequencing get the 3rd reads

Data analysis

Software used are listed as follows:
CANU 1.5
BWA : version 0.7.17
SMRT analysis software (v2.3.1)
SSPACE v3.0
GATK v2.7-2
joinMAP v4.1
MSTmap v1.0
NUCmer: MUMmer Package v3.23
BLASTP : ncbi-BLAST v2.2.28
BLASTN : ncbi-BLAST v2.2.28
Cufflinks v2.2.1
LTR_FINDER v1.0.2
RepeatModeler (v1.0.3)
RepeatMasker (v3.2.9)

Tandem Repeats Finder (TRF, v4.04)
LTRharvest (genometools, v1.5.6)
ClustalW (v2.1)
PAML (v4.8)
hisat2 (v2.0.4)
Stringtie (v 1.2.3)
tRNAscan-SE (v 1.23)
infernal (version 1.0)
Gramene pipeline (v 1.0)
SOAP-trans v1.03
InterProScan v5.27
OrthoMCL v2.0.9
Trimmomatic v0.35
Bowtie 2 v2.2.6
RSEM (v1.2.25)
edgeR (v3.6)
Trinity (v2.1.0)
Trinotate (v2.0.2)
SOAP.COVERAGE v2.7.7
MCScanX v1.0
ngsShoRT v2.2
TopHat2 v2.0.10
SAMtools v0.1.20
MEGA version 5

For manuscripts utilizing custom algorithms or software that are central to the research but not yet described in published literature, software must be made available to editors/reviewers upon request. We strongly encourage code deposition in a community repository (e.g. GitHub). See the Nature Research guidelines for submitting code & software for further information.

Data

Policy information about availability of data

All manuscripts must include a data availability statement. This statement should provide the following information, where applicable:

- Accession codes, unique identifiers, or web links for publicly available datasets
- A list of figures that have associated raw data
- A description of any restrictions on data availability

Genome assembly data have been deposited at NCBI BioProject ID under accession codes PRJNA227267. The raw sequence data reported in this paper have been deposited in the Genome Sequence Archive103 in BIG Data Center104, Beijing Institute of Genomics (BIG), Chinese Academy of Sciences, under accession numbers PRJCA000223 and PRJCA001050 that are publicly accessible at http://bigd.big.ac.cn/gsa. We built the Antirrhinum genome website at http://bioinfo.sibs.ac.cn/Am, providing a portal to genome browser, Blast, data download and gene expression functions. All data that support the findings of this study are also available from the corresponding authors upon request.

Field-specific reporting

Please select the best fit for your research. If you are not sure, read the appropriate sections before making your selection.

☒ Life sciences ☐ Behavioural & social sciences ☐ Ecological, evolutionary & environmental sciences

For a reference copy of the document with all sections, see nature.com/authors/policies/ReportingSummary-flat.pdf

Life sciences study design

All studies must disclose on these points even when the disclosure is negative.

Sample size

A total of 48 RILs were used for linkage map construction. Line 337.

Data exclusions

Line 386-401：
1.The raw reads generated from the Illumina-Pipeline included low-quality, adapter contaminated and duplicated reads. Reads were filtered using Trimmomatic50 with default parameters.
2.SAMtools52 to filter out low-quality (mapping quality <30) alignments
3.filtered using the GATK VariantFiltration program with the following criteria: clusterWindowSize:10, MQ0>=4& ((MQ0/ (1.0* DP)) >0.1), QUAL<50.0, DP< 5.
4. filter out unmatched SNPs and not homozygous for parents

Line 438:mRNAs and ESTs of eudicot species were downloaded from NCBI and filtered to remove redundant sequences with a cutoff of 90% for both identity and coverage,
Line459:the CPC program71 and gene prediction evidence such as poor coding ability and protein length were used to filter the noncoding genes.
Line 533:BLASTP and a filter threshold of 1e-5

Replication	Three experiments were performed in Fluorescence In Situ Hybridization (FISH). Line 572.
Randomization	No randomization is required for our experiments.
Blinding	Blind experiment is not required for our work.

Behavioural & social sciences study design

All studies must disclose on these points even when the disclosure is negative.

Study description	*Briefly describe the study type including whether data are quantitative, qualitative, or mixed-methods (e.g. qualitative cross-sectional, quantitative experimental, mixed-methods case study).*
Research sample	*State the research sample (e.g. Harvard university undergraduates, villagers in rural India) and provide relevant demographic information (e.g. age, sex) and indicate whether the sample is representative. Provide a rationale for the study sample chosen. For studies involving existing datasets, please describe the dataset and source.*
Sampling strategy	*Describe the sampling procedure (e.g. random, snowball, stratified, convenience). Describe the statistical methods that were used to predetermine sample size OR if no sample-size calculation was performed, describe how sample sizes were chosen and provide a rationale for why these sample sizes are sufficient. For qualitative data, please indicate whether data saturation was considered, and what criteria were used to decide that no further sampling was needed.*
Data collection	*Provide details about the data collection procedure, including the instruments or devices used to record the data (e.g. pen and paper, computer, eye tracker, video or audio equipment) whether anyone was present besides the participant(s) and the researcher, and whether the researcher was blind to experimental condition and/or the study hypothesis during data collection.*
Timing	*Indicate the start and stop dates of data collection. If there is a gap between collection periods, state the dates for each sample cohort.*
Data exclusions	*If no data were excluded from the analyses, state so OR if data were excluded, provide the exact number of exclusions and the rationale behind them, indicating whether exclusion criteria were pre-established.*
Non-participation	*State how many participants dropped out/declined participation and the reason(s) given OR provide response rate OR state that no participants dropped out/declined participation.*
Randomization	*If participants were not allocated into experimental groups, state so OR describe how participants were allocated to groups, and if allocation was not random, describe how covariates were controlled.*

Ecological, evolutionary & environmental sciences study design

All studies must disclose on these points even when the disclosure is negative.

Study description	*Briefly describe the study. For quantitative data include treatment factors and interactions, design structure (e.g. factorial, nested, hierarchical), nature and number of experimental units and replicates.*
Research sample	*Describe the research sample (e.g. a group of tagged Passer domesticus, all Stenocereus thurberi within Organ Pipe Cactus National Monument), and provide a rationale for the sample choice. When relevant, describe the organism taxa, source, sex, age range and any manipulations. State what population the sample is meant to represent when applicable. For studies involving existing datasets, describe the data and its source.*
Sampling strategy	*Note the sampling procedure. Describe the statistical methods that were used to predetermine sample size OR if no sample-size calculation was performed, describe how sample sizes were chosen and provide a rationale for why these sample sizes are sufficient.*
Data collection	*Describe the data collection procedure, including who recorded the data and how.*
Timing and spatial scale	*Indicate the start and stop dates of data collection, noting the frequency and periodicity of sampling and providing a rationale for these choices. If there is a gap between collection periods, state the dates for each sample cohort. Specify the spatial scale from which the data are taken*
Data exclusions	*If no data were excluded from the analyses, state so OR if data were excluded, describe the exclusions and the rationale behind them, indicating whether exclusion criteria were pre-established.*
Reproducibility	*Describe the measures taken to verify the reproducibility of experimental findings. For each experiment, note whether any attempts to repeat the experiment failed OR state that all attempts to repeat the experiment were successful.*
Randomization	*Describe how samples/organisms/participants were allocated into groups. If allocation was not random, describe how covariates were controlled. If this is not relevant to your study, explain why.*

Blinding	*Describe the extent of blinding used during data acquisition and analysis. If blinding was not possible, describe why OR explain why blinding was not relevant to your study.*

Did the study involve field work? ☐ Yes ☒ No

Field work, collection and transport

Field conditions	*Describe the study conditions for field work, providing relevant parameters (e.g. temperature, rainfall).*
Location	*State the location of the sampling or experiment, providing relevant parameters (e.g. latitude and longitude, elevation, water depth).*
Access and import/export	*Describe the efforts you have made to access habitats and to collect and import/export your samples in a responsible manner and in compliance with local, national and international laws, noting any permits that were obtained (give the name of the issuing authority, the date of issue, and any identifying information).*
Disturbance	*Describe any disturbance caused by the study and how it was minimized.*

Reporting for specific materials, systems and methods

Materials & experimental systems

n/a	Involved in the study
☒	☐ Unique biological materials
☒	☐ Antibodies
☒	☐ Eukaryotic cell lines
☒	☐ Palaeontology
☒	☐ Animals and other organisms
☒	☐ Human research participants

Methods

n/a	Involved in the study
☒	☐ ChIP-seq
☒	☐ Flow cytometry
☒	☐ MRI-based neuroimaging

Unique biological materials

Policy information about availability of materials

Obtaining unique materials	No unique materials in this study.

Antibodies

Antibodies used	No antibody was used in this study.
Validation	No antibody was used in this study.

Eukaryotic cell lines

Policy information about cell lines

Cell line source(s)	No eukaryotic cell line was used in this study.
Authentication	No eukaryotic cell line was used in this study.
Mycoplasma contamination	No eukaryotic cell line was used in this study.
Commonly misidentified lines (See ICLAC register)	No eukaryotic cell line was used in this study.

Palaeontology

Specimen provenance	No palaeontolog`s materials was used in this study.
Specimen deposition	No palaeontolog`s materials was used in this study.

Dating methods | No palaeontolog`s materials was used in this study.

☐ Tick this box to confirm that the raw and calibrated dates are available in the paper or in Supplementary Information.

Animals and other organisms

Policy information about studies involving animals; ARRIVE guidelines recommended for reporting animal research

Laboratory animals	no animals` data was used in this study.
Wild animals	no animals` data was used in this study.
Field-collected samples	no animals` was used in this study.

Human research participants

Policy information about studies involving human research participants

Population characteristics	No human`s data was used in this study
Recruitment	No human`s data was used in this study

ChIP-seq

Data deposition

☐ Confirm that both raw and final processed data have been deposited in a public database such as GEO.

☐ Confirm that you have deposited or provided access to graph files (e.g. BED files) for the called peaks.

Data access links<br>*May remain private before publication.*	No ChIP-seq used in this study.
Files in database submission	No ChIP-seq used in this study.
Genome browser session<br>(e.g. UCSC)	No ChIP-seq used in this study.

Methodology

Replicates	No Methodology used in this study.
Sequencing depth	No Methodology used in this study.
Antibodies	No Methodology used in this study.
Peak calling parameters	No Methodology used in this study.
Data quality	No Methodology used in this study.
Software	No Methodology used in this study.

Flow Cytometry

Plots

Confirm that:

☐ The axis labels state the marker and fluorochrome used (e.g. CD4-FITC).

☐ The axis scales are clearly visible. Include numbers along axes only for bottom left plot of group (a 'group' is an analysis of identical markers).

☐ All plots are contour plots with outliers or pseudocolor plots.

☐ A numerical value for number of cells or percentage (with statistics) is provided.

Methodology

Sample preparation	No flow cytometry used in this study.
Instrument	No flow cytometry used in this study.

Software	No flow cytometry used in this study.
Cell population abundance	No flow cytometry used in this study.
Gating strategy	No flow cytometry used in this study.

☐ Tick this box to confirm that a figure exemplifying the gating strategy is provided in the Supplementary Information.

Magnetic resonance imaging

Experimental design

Design type	No Magnetic resonance imaging used in this study
Design specifications	No Magnetic resonance imaging used in this study
Behavioral performance measures	No Magnetic resonance imaging used in this study

Acquisition

Imaging type(s)	No Magnetic resonance imaging used in this study
Field strength	No Magnetic resonance imaging used in this study
Sequence & imaging parameters	No Magnetic resonance imaging used in this study
Area of acquisition	No Magnetic resonance imaging used in this study

Diffusion MRI ☐ Used ☒ Not used

Preprocessing

Preprocessing software	No Magnetic resonance imaging used in this study
Normalization	No Magnetic resonance imaging used in this study
Normalization template	No Magnetic resonance imaging used in this study
Noise and artifact removal	No Magnetic resonance imaging used in this study
Volume censoring	No Magnetic resonance imaging used in this study

Statistical modeling & inference

Model type and settings	No Magnetic resonance imaging used in this study
Effect(s) tested	No Magnetic resonance imaging used in this study

Specify type of analysis: ☐ Whole brain ☐ ROI-based ☐ Both

Statistic type for inference (See Eklund et al. 2016)	No Magnetic resonance imaging used in this study
Correction	No Magnetic resonance imaging used in this study

Models & analysis

n/a	Involved in the study
☒	☐ Functional and/or effective connectivity
☒	☐ Graph analysis
☒	☐ Multivariate modeling or predictive analysis

Functional and/or effective connectivity	*Report the measures of dependence used and the model details (e.g. Pearson correlation, partial correlation, mutual information).*
Graph analysis	*Report the dependent variable and connectivity measure, specifying weighted graph or binarized graph, subject- or group-level, and the global and/or node summaries used (e.g. clustering coefficient, efficiency, etc.).*
Multivariate modeling and predictive analysis	*Specify independent variables, features extraction and dimension reduction, model, training and evaluation metrics.*
